# The Activity of Novel BCR-ABL Small-Molecule Degraders Containing Pyrimidine Rings and Their Role in Overcoming Drug Resistance

**DOI:** 10.1155/2022/4056398

**Published:** 2022-10-30

**Authors:** Xu Zhang, Linglan Tu, Haohuan Chai, Zilin Li, Yuhan Fu, Xiaoliang Zheng, Shenxin Zeng, Liyan Cheng

**Affiliations:** ^1^School of Laboratory Medicine and Bioengineering, Hangzhou Medical College, Hangzhou, Zhejiang, China; ^2^Zhejiang Provincial Laboratory of Experimental Animal's and Nonclinical Laboratory Studies, Hangzhou, Zhejiang, China; ^3^School of Pharmacy, Hangzhou Medical College, Hangzhou, Zhejiang, China

## Abstract

Inducing protein degradation by proteolysis-targeting chimeras (PROTACs) has gained tremendous momentum in the field for its promise in the discovery and development of new therapies. Based on our previously reported PROTAC BCR-ABL degraders, we designed and synthesized additional 4 PROTAC compounds with a novel linker that contains pyrimidine rings. Molecular and cellular studies have shown that different linkers affect the degradation activity of small-molecule degraders on the target protein of BCR-ABL. We screened out a lead compound, **DMP11**, with stable physicochemical properties and high activity. Preliminary evaluation of its pharmacodynamics in vitro model showed that it has a good inhibitory effect on imatinib-resistant chronic myeloid leukemia cell lines, as has been shown in animal models. Our preliminary research into the mechanism of **DMP11** found that **DMP11** can overcome drug resistance by simultaneously inhibiting the targets of BCR-ABL and SRC-family kinase (SFK).

## 1. Introduction

BCR-ABL is both necessary and sufficient for the development of CML. Due to the advent of tyrosine kinase inhibitors, the cure rate of CML has improved greatly [1–4]. Unfortunately, a subset of CML patients develop resistance to TKIs, leading to treatment failure and progression to blast phase CML (BP-CML) [5–9]. Despite advances in BCR-ABL-targeted therapy, stem cell transplantation remains the only treatment option for BP-CML [10, 11]. Current research frontiers in CML include overcoming TKI resistance, improving the prognosis in BP-CML, and increasing treatment-free remission (TFR) rates, which are active areas of research in CML [12–15].

PROTAC achieves biological activity in an event-driven mode of action, fundamentally reversible and rapid knockdown of pathogenic proteins, which can affect both kinase-dependent and -independent biological functions. Theoretically, it can delay or even eliminate drug resistance. PROTAC technology may provide a new strategy and option for clinical targeted tumor therapy. It also provides new ideas for addressing the abovementioned problems, such as drug resistance and improving the prognosis of BP-CML. A series of studies have found that small-molecule degraders can reduce drug resistance. They also have little off-target toxicity [16–20]. Our previous article summarized the recent progress and challenges faced by PROTACs in the field of drug development [21]. On the basis of previous studies, we explored various structures and prepared a lead compound, DMP11, a linker containing a pyrimidine ring with stable physicochemical properties and high activity. As a “privileged scaffold,” the pyrimidine ring is widely used in the field of new drug research and development, and its unique mode of action with the receptor greatly improves the selectivity of drug molecules and anticancer activity and can reduce toxic and side effects [22, 23]. PROTAC can effectively inhibit the target protein at a low dose and quickly degrade and clear it, providing an efficient strategy with high safety, antidrug resistance, and broad application prospects [24].

## 2. Materials and Methods

### 2.1. Compound Synthesis

The commercially available reagents and solvents were used as purchased without further purification. When needed, solvents were distilled and stored on molecular sieves. Column chromatography was performed on silica gel. Thin layer chromatography (TLC) was carried out on 5 cm × 20 cm plates with a layer thickness of 0.25 mm. When necessary, TLC plates were visualized with aqueous KMnO_4_ or with an aqueous Pancaldi solution. All the target compounds were checked by ^1^HNMR (Bruker Avance Neo 400 MHz), ^13^C-NMR (Bruker Avance Neo 100 MHz), and mass spectrometry (Waters Q-Tof) equipped with an ESI source and an ion trap detector. Chemical shifts are reported in parts per million (ppm).

Synthetic route of the target compound: intermediates **5–8** were prepared according to our previous report.

  N-(2-Chloro-6-methylphenyl)-2-((6-(4-(5-((6-((2-(2,6-dioxopiperidin-3-yl)-1-oxoisoindolin-4-yl) amino)-6-oxohexyl)carbamoyl)pyrimidin-2-yl)piperazin-1-yl)-2-methylpyrimidin-4-yl)amino)thiazole-5-carboxamide (DMP 6) to a solution of compound **4** (200 mg, 0.354 mmol, 1 eq) in DMF (25 mL), DIPEA (136.74 mg, 1.06 mmol, 3 eq), and HATU (204.03 mg, 25.39 mmol, 1.5 eq) were added in order. The reaction mixture was stirred at room temperature for 30 min. Then, compound **5** was added to continue the reaction at ambient temperature and maintain it for 6 h. Next, the reaction mixture was quenched by the addition of H_2_O (200 mL) and extracted with EtOAc (200 mL × 3). The organic extract was dried over Na_2_SO_4_, filtered, and concentrated in vacuo. Purification by silica gel column chromatography using DCM/EA 50 : 1 provided the desired compound (105 mg, 0.114 mmol, 32.2%). ^1^H NMR (400 MHz, DMSO) *δ* 11.52 (s, 1H), 11.03 (s, 1H), 9.90 (s, 1H), 9.77 (s, 1H), 8.81 (s, 2H), 8.34 (t, *J* = 5.4 Hz, 1H), 8.24 (s, 1H), 7.82 (dd, *J* = 7.3, 1.4 Hz, 1H), 7.61–7.45 (m, 2H), 7.40 (dd, *J* = 7.3, 1.4 Hz, 1H), 7.32-7.13 (m, 2H), 6.12 (s, 1H), 5.16 (dd, *J* = 13.3, 5.1 Hz, 1H), 4.38 (q, *J* = 17.5 Hz, 2H), 3.93 (t, *J* = 5.3 Hz, 4H), 3.75–3.61 (m, 4H), 3.24 (dd, *J* = 12.6, 6.4 Hz, 2H), 2.99-2.83 (m, 1H), 2.68-2.56 (m, 1H), 2.45 (s, 3H), 2.40-2.30 (m, 3H), 2.25 (s, 3H), 2.03 (dd, *J* = 8.8, 3.7 Hz, 1H), 1.70–1.57 (m, 2H), 1.56-1.47 (m, 2H), 1.37-1.27 (m, 6H); ^13^C NMR (100 MHz, DMSO) *δ* 173.3, 171.9, 171.6, 168.3, 165.7, 163.9, 163.0, 162.7, 161.8, 160.4, 158.0, 157.4, 141.3, 139.3, 134.3, 134.2, 133.9, 133.1, 132.9, 129.5, 129.1, 128.6, 127.5, 126.2, 125.7, 119.5, 117.0, 83.2, 52.0, 46.9, 43.6, 43.3, 40.6, 40.3, 40.1, 36.2, 31.7, 29.6, 29.1, 29.0, 26.8, 26.1, 25.5, 23.1, 18.8. MS (ESI^+^): m/z 920.3175 [M + H]^+^.
  N-(2-Chloro-6-methylphenyl)-2-((6-(4-(5-((8-((2-(2,6-dioxopiperidin-3-yl)-1-oxoisoindolin-4-yl) amino)-8-oxooctyl) carbamoyl) pyrimidin-2-yl) piperazin-1-yl)-2-methylpyrimidin-4-yl) amino) thiazole-5-carboxamide (DMP 7).
  DMP 7 was condensed from **4** and **6** according to the general method (130 mg, 0.137 mmol, 38.7%). ^1^H NMR (400 MHz, DMSO) *δ* 11.52 (s, 1H), 11.03 (s, 1H), 9.90 (s, 1H), 9.77 (s, 1H), 8.81 (s, 2H), 8.34 (t, *J* = 5.5 Hz, 1H), 8.24 (s, 1H), 7.82 (dd, *J* = 7.2, 1.6 Hz, 1H), 7.54-7.44 (m, 2H), 7.42–7.38 (m, 1H), 7.32-7.21 (m, 2H), 6.12 (s, 1H), 5.15 (dd, *J* = 13.3, 5.1 Hz, 1H), 4.37 (q, *J* = 17.5 Hz, 2H), 3.93 (d, *J* = 5.5 Hz, 4H), 3.72-3.63 (m, 4H), 3.25-3.16 (m, 2H), 3.00-2.84 (m, 1H), 2.65-2.57 (m, 1H), 2.44 (s, 3H), 2.40-2.32 (m, 3H), 2.25 (s, 3H), 2.09-1.98 (m, 1H), 1.64-1.56 (m, 2H), 1.53–1.46 (m, 2H), 1.29-1.21 (m, 18H); ^13^C NMR (100 MHz, DMSO) *δ* 173.3, 171.8, 171.5, 168.3, 165.6, 163.8, 163.0, 162.8, 161.8, 160.4, 158.0, 157.4, 141.3, 139.3, 134.3, 134.1, 134.0, 133.1, 129.3, 129.1, 127.5, 126.2, 125.7, 119.4, 117.0, 83.2, 52.0, 46.9, 43.5, 43.3, 36.3 31.6, 30.2, 29.6, 29.4, 29.2, 29.1, 26.9, 26.0, 25.6, 23.1, 22.6, 18.7, 14.4. MS (ESI^+^): m/z 948.3490 [M + H]^+^.
  N-(2-Chloro-6-methylphenyl)-2-((6-(4-(5-((11-((2-(2,6-dioxopiperidin-3-yl)-1-oxoisoindolin-4-yl) amino)-11-oxoundecyl) carbamoyl) pyrimidin-2-yl) piperazin-1-yl)-2-methylpyrimidin-4-yl) amino) thiazole-5-carboxamide (DMP 11).
  DMP 11 was condensed from 4 to 7 according to the general method (125 mg, 0.126 mmol, 35.6%). ^1^H NMR (400 MHz, DMSO) *δ* 11.50 (s, 1H), 11.02 (s, 1H), 9.88 (s, 1H), 9.77 (s, 1H), 8.79 (s, 2H), 8.36 (t, *J* = 5.5 Hz, 1H), 8.24 (s, 1H), 7.81 (dd, *J* = 7.5, 1.2 Hz, 1H), 7.51 (dd, *J* = 7.5, 1.2 Hz, 1H), 7.49-7.44 (m, 1H), 7.39 (t, *J* = 7.4, 1.7 Hz, 1H), 7.30-7.26 (m, 1H), 7.27-7.21 (m, 1H), 6.12 (s, 1H), 5.15 (dd, *J* = 13.3, 5.1 Hz, 1H), 4.47-4.26 (m, 2H), 4.03-3.87 (m, 4H), 3.73-3.63 (m, 4H), 3.28-3.22 (m, 2H), 3.09 (qd, *J* = 7.3, 4.8 Hz, 1H), 2.98-2.82 (m, 1H), 2.67-2.57 (m, 1H), 2.45 (s, 3H), 2.41-2.33 (mz, 2H), 2.26 (s, 3H), 2.08-2.00 (m, 1H), 1.71-1.62 (m, 2H), 1.61-1.53 (m, 2H), 1.45-1.35 (m, 2H), 1.28-1.15 (m, 4H); ^13^C NMR (100 MHz, DMSO) *δ* 173.2, 171.8, 171.5, 168.3, 165.6, 163.9, 163.1, 162.7, 161.8, 160.4, 157.9, 157.5, 141.3, 139.3, 134.2, 134.1, 134.0, 133.1, 132.9, 129.4, 128.9, 128.5, 127.4, 126.2, 125.7, 119.4, 117.0, 83.3, 79.7, 79.4, 79.1, 55.6, 52.0, 46.9, 46.1, 43.6, 43.3, 36.2, 31.7, 29.4, 26.5, 26.0, 25.3, 23.1, 18.8, 9.0. MS (ESI^+^): m/z 990.4318 [M + H]^+^.
  N-(2-Chloro-6-methylphenyl)-2-((6-(4-(5-((12-((2-(2,6-dioxopiperidin-3-yl)-1-oxoisoindolin-4-yl)amino)-12-oxododecyl)carbamoyl)pyrimidin-2-yl)piperazin-1-yl)-2-methylpyrimidin-4-yl) amino) thiazole-5-carboxamide (DMP 12).
  The DMP 12 was condensed from **4** and **8** according to the general method (150 mg, 0.150 mmol, 42.4%). ^1^H NMR (400 MHz, DMSO) *δ* 11.51 (s, 1H), 11.03 (s, 1H), 9.90 (s, 1H), 9.78 (s, 1H), 8.81 (s, 2H), 8.35 (t, *J* = 5.5 Hz, 1H), 8.25 (s, 1H), 7.82 (dd, *J* = 7.2, 1.6 Hz, 1H), 7.53–7.47 (m, 2H), 7.40 (dd, *J* = 7.2, 1.6 Hz, 1H), 7.31–7.25 (m, 2H), 6.12 (s, 2H), 5.16 (dd, *J* = 13.3, 5.1 Hz, 1H), 4.39–4.35 (m, 2H), 3.93 (d, *J* = 5.5 Hz, 6H), 3.70-3.64 (m, 4H), 3.25-3.11 (m, 2H), 3.09-2.82 (m, 2H), 2.70-2.54 (m, 2H), 2.45 (s, 3H), 2.36-2.34 (m, 3H), 2.25 (s, 3H), 2.08-1.95 (m, 1H), 1.70-1.57 (m, 2H), 1.50–1.47 (m, 4H), 1.36-1.21 (m, 14H); ^13^C NMR (101 MHz, DMSO) *δ* 173.3, 171.8, 171.5, 168.3, 165.6, 163.8, 163.0, 162.7, 161.8, 160.4, 158.0, 157.4, 141.3, 139.3, 134.3, 134.1, 134.0, 133.1, 132.9, 129.5, 129.1, 128.6, 127.4, 126.2, 125.7, 123.2, 119.4, 117.0, 116.0, 107.4, 97.6, 83.2, 68.7, 67.1, 52.0, 46.9, 46.1, 43.6, 43.3, 36.2, 34.8, 32.0, 31.6, 29.9, 29.6, 29.5, 29.4, 29.2, 29.1, 27.8, 26.9, 26.0, 25.5, 24.0, 23.1, 22.2, 18.7, 9.3. MS (ESI^+^): m/z 1004.4087 [M + H]^+^.

### 2.2. Cell Lines and Cell Culture

K562 cell lines were laboratory-owned, and KA (imatinib-resistant K562) was purchased from the Shanghai Institute of Cell Biology, Chinese Academy of Sciences. All the cells were cultured in RPMI-1640 (Gibco, Grand Island, New York, USA) containing 10% fetal bovine serum (FBS; HyClone, Logan, UT, USA) at 37°C with 95% humidity and 5% CO_2_. The medium was changed every 2-3 days. All the cells were cultured according to the instructions.

### 2.3. Cell Viability Assay and Measurement of Dose-Effect Relationship

Cell viability was assessed by the Cell Titer-Glo® luminescent cell viability assay. K562 and KA cells in the logarithmic growth phase were inoculated into 96-well plates with a cell density of 1 × 10^5^/well and incubated at 37°C for 24 hours. Then, each test drug was set into 5 concentration gradients, and the cell-free medium was used as the blank control and was added into the well plate. Four multiple wells were set for each concentration. The drug was administered at 37°C for 72 hours; Put Cell Titer-Glo®'s reagent at room temperature until it dissolves, mix it according to the instructions, add the same volume to the test well plate, then place it on the oscillator and shake it for 2 minutes to induce cell lysis, leave it at room temperature for 10 minutes to stabilize the luminescence signal, and then measure the absorbance value of each well with a microplate reader. GraphPad Prism6 statistically analyzed the dose-effect relationship of tested drugs and calculated the IC_50_.

### 2.4. Measurement of Time Effect Relationship

K562 and KA cells in the logarithmic growth phase were inoculated into 96-well plates with a cell density of 1 × 10^5^/well and incubated at 37°C for 24 hours. After determining the dose of the tested drug according to IC_50_, set different time points of action between the tested drug and the cell, and set 4 multiple holes for each time point. At the end of the time point, add the Cell Titer-Glo® reagent in equal volume to the test well plate, and then shock it on the oscillator for 2 minutes to induce cell lysis. The luminescence signal was stable after being placed at room temperature for 10 minutes. Then the absorbance value of each well was measured with a microplate reader, and the aging relationship of the tested drugs was statistically analyzed by GraphPad Prism6.

### 2.5. Western Blot

K562 and KA were treated with different concentrations of **DMP** or at different time points. After adding the lysate (50 mM NaCl, 5 mM EDTA, 0.5% SDS, 0.1 mM sodium orthovanadate, 50 *μ*g/mL aprotinin, 1 mM phenylmethysulfonyl fluoride, and 10 mM Tris-HCl; pH 7.4), shock lysis was performed on the ice bath for 30 min, followed by ultrasonic nucleation on the ice bath for 30 seconds (50% strength, 2 s/4 s), centrifugation at 12000 rpm at 4°C for 15 min. After the supernatant was taken, the protein was quantified and SDS-PAGE gel electrophoresis was performed. After electrophoresis, the samples were transferred to nitrocellulocellulose membrane, followed by an immune reaction, and then incubated with c-Abl antibody (Cell Signaling Technology #2862), SRC antibody (Cell Signaling Technology #2108), *β*-actin (Cell Signaling Technology #8457) at room temperature for 4°C overnight. Goat anti-rabbit IgG-HRP (Cell Signaling Technology) was incubated for 2 hours, TBST was washed for 2 hours, and ECL solution was added for 1 minute. The membrane was drained and exposed in a bio-RAD chemiluminescence imager for several minutes. The results were read by ImageLab 5.2.1 software and analyzed statistically with *β*-actin as the internal reference.

### 2.6. Lentivirus Transfection to Construct Luciferase-Labeled K562 and KA Cell Lines

The cells were centrifuged in a 1.5 mL EP tube and then diluted with 100 *μ*L of serum-free medium to completely submerge the cells in the medium. According to the number of virus particles required by MOI, the virus solution was inhaled into cells and a 1.5 mL EP tube was incubated at 37°C for 30 min. The mixed solution was sucked out of the test tube and added into the Petri dish medium. Enough fresh medium was added and 6 *μ*g/mL Polybrene was added to improve the transfection efficiency. After 48 h, the medium was changed to increase the concentration of purinomycin, and positive cells were screened. The positive rate of cells was observed after 96 h.

### 2.7. Luciferase Reporter Assay

A luciferase reporter test was carried out in a luciferase reporter assay system (Promega). The targeted cells of K562 and KA were incorporated into 30 ng luciferase reporter vectors. Luciferase activity was determined as per the manufacturer's instructions 24 h after transfection.

### 2.8. In Situ Model Establishment and Tumor Cell Measurement Methods

The animal studies were approved by the Zhejiang Experimental Animal Center and were carried out according to institutional guidelines. The density of luciferase-transfected cells in NSG (NOD/SCID*γ*Cnull) male mice was 1 × 10^7^/mL after 6 weeks of injection, after inoculation on days 3, 7, 14, 21, and 28, live performance imaging mice using NightOWL II 983-pound live animal visible-light imaging system were observed for nested, invasive metastases.

### 2.9. Quantifification and Statistical Analysis

All the cell data of protein expression assay and cell growth inhibition assay were presented as mean ± standard error from at least three independent experiments. Statistical analyses presented in all figures were performed using GraphPad Prism software (version 8.00).

## 3. Result

### 3.1. Design of DMP11 as a New and Bona Fide PROTAC BCR-ABL Degrader

**Figure 1:**
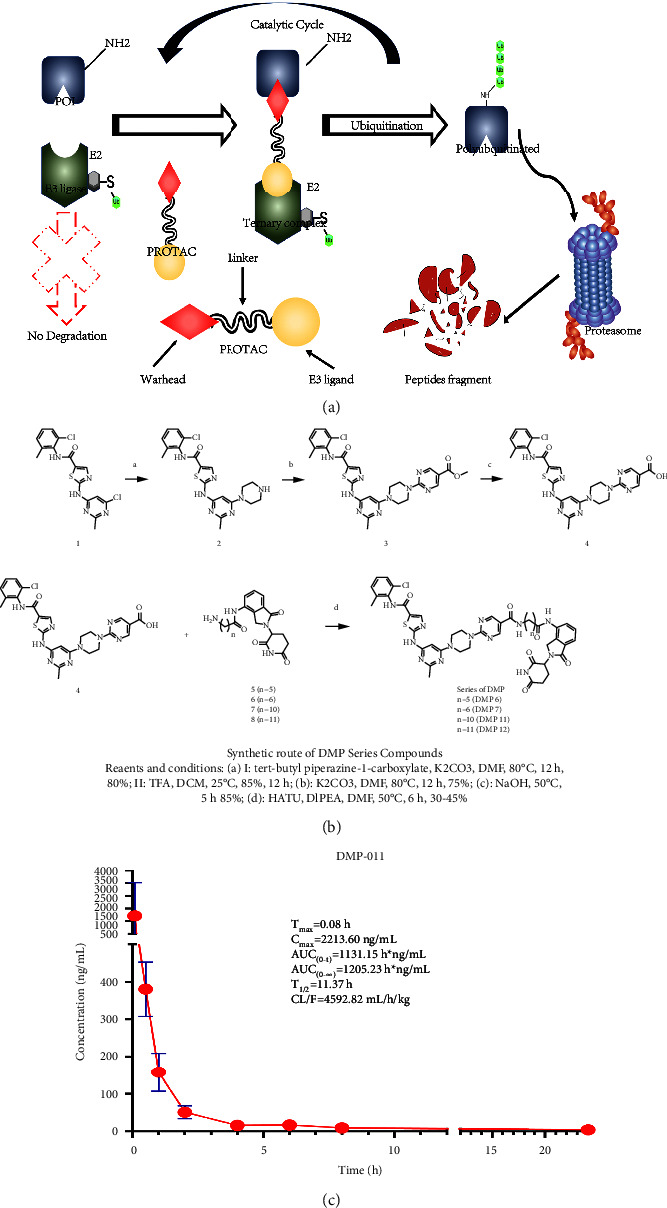
Synthesis and pharmacokinetics of protacs targeting BCR-ABL. (a) A PROTAC molecule consists of a warhead to target the POI. An E3 ligand to recruit the E3 ligase and a linker to tether them together. (b) Synthesis of protacs compounds targeting BCR-ABL degradation. (c) Pharmacokinetic results of DMP11. Three biological replicates of each treatment were performed and the data are presented as the mean ± SD.

Target compound synthesis: PROTAC consists of three parts (Figure 1(a)). First, we synthesized the target protein-ligand and linker and then exposed the active functional group and spliced the product with the E3 ligase ligand. Next, we covalently linked the E3 ligase to the linker and finally connected the target protein ligands. All series used commercialized and inexpensive lenalidomide and dasatinib derivatives (compound 3) as starting materials to synthesize target molecules. The 2nd position of ethyl 2-chloropyrimidine-5-carboxylate is attacked by Boc piperazine under alkaline conditions. Similarly, compounds 2 and 3 are nucleophilically substituted under alkaline conditions, and the ester bonds are hydrolyzed in NaOH solution to produce compound 4. The condensation of the amino group and carboxyl group forms an amide bond that includes the common condensing agent HATU. This synthetic route is widely used in the synthesis of PROTAC and polypeptide chains as it is convenient to perform and has a high yield. All compounds were synthesized by our laboratory and the purity was greater than 96% (Figure 1(b)) . In order to investigate whether the **DMP** series compounds have good pharmacokinetic behavior, we administered **DMP-11** to rats by tail vein injection (5 mg/kg) to investigate the pharmacokinetic behavior. The experimental results showed that the compound **DMP-11** was not absorbed by intragastric administration and had good pharmacokinetic behavior after intravenous administration (Figure 1(c)).

### 3.2. DMP11 Shows a Highly Potent Inhibitory Effect on Imatinib-Resistant Chronic Myeloid Leukemia

**Figure 2:**
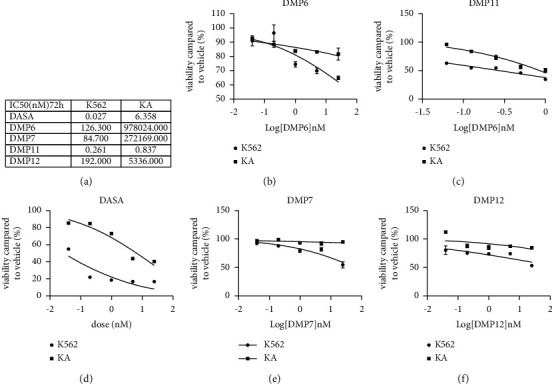
Effects of different compounds on cell viability and IC_50_ in imatinib-resistant cell lines and wild-type cell lines in CML. (a) Linker exploration between dasatinib and lenalidomide moiety and the corresponding IC_50_ in the K562 and KA. (b, c, d, e, f) viability of K562 and KA cells after incubation with a range of compound concentrations for 72 h as determined by CellTiter-Glo® reagent assay. Three biological replicates of each treatment were performed, and the data are presented as the mean ± SD.

The four compounds with good binding potency screened by our fluorescence polarization (FP)-based binding assay were subjected to cell activity inhibition experiments. We compared different linker lengths in the chronic myeloid leukemia cell line K562 and the drug-resistant strain KA. The effect of degraders targeting the degradation of BCR-ABL protein on the cell viability of these two cell lines was recorded. Results showed that a **DMP11** with a linker of 10 carbons could significantly inhibit the cell activity of K562 and the imatinib resistance KA with an IC_50_ of 0.261 nM and 0.837 nM, respectively (Figure 2(a)). Similarly, **DMP6**, **DMP7**, and **DMP12** with 5, 6, and 11 carbon linkers using dasatinib as the warhead did not show any obvious inhibitory effect on the imatinib-resistance cell line KA (Figures 2(b)–2(f).

### 3.3. DMP11 Degrades BCR-ABL and Src Protein in a Dose and Time-Dependent Manner

**Figure 3:**
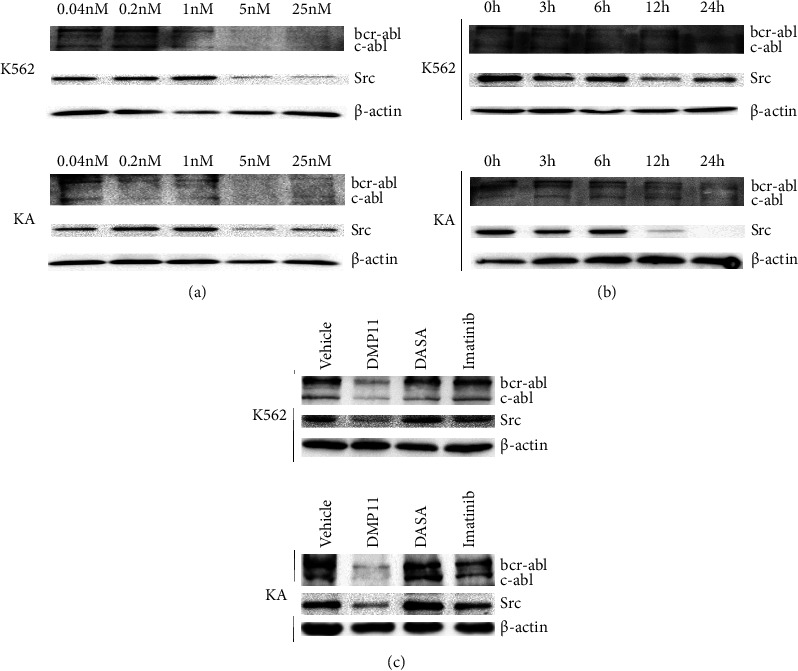
The targeted degradation of DMP11 on BCR-ABL and SRC were observed at different doses and at different time points. (a) Dose-dependent degradation of BCR-ABL and SRC proteins by DMP11 in K562 and KA cell lines. (b) Time-dependent degradation of BCR-ABL and SRC proteins by DMP11 in K562 and KA cell lines. Three biological replicates of each treatment were performed. (c) Comparison of the degradation of BCR-ABL and Src proteins by DMP11 (5 nM), DASA (5 nM), and imatinib (5 nM) for 24 h. Three biological replicates of each treatment were performed.

We observed the targeted degradation of the fusion protein BCR-ABL by **DMP11** at different doses and at different times. Results showed that **DMP11** at a dose of 5 nM significantly degraded the BCR-ABL fusion protein of wild-type and imatinib-resistant chronic myeloid leukemia cell lines and found that the SRC protein (Yes/Fyn/Fgr) also showed a dose-dependent degradative effect (Figure 3(a)). When wild-type and drug-resistant chronic myeloid leukemia cells were incubated with **DMP11** for 24 hours, the fusion protein became significantly degraded, and the SRC protein (Yes/Fyn/Fgr) was also degraded (Figure 3(b)). However, dasatinib and imatinib had almost no degradation effect on BCR-ABL and Src protein under the same incubation time and dose (Figure 3(c)).

### 3.4. Degradation of SRC Proteins Is Not Achieved by Downregulation of BCR-ABL but by Degradation of Lysosomes

**Figure 4:**
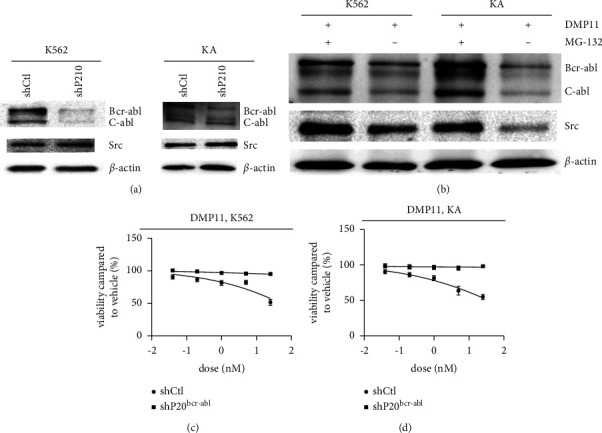
Degradation of SRC proteins is not achieved by downregulation of BCR-ABL, but by degradation of lysosomes. (a) Lentivirus-packed plasmids knock down expression of the BCR-ABL fusion protein in K562 and KA cell lines. (b) Western blot analysis of P210 and Src levels in K562 and KA cell lines after the cotreatment with 10 *μ*M MG132 and 5 nM DMP11 for 24 h (c) After knocking down P210, the dose-dependent inhibition of DMP11 on K562 cell line disappeared. (d) After knocking down P210, the dose-dependent inhibitory effect of DMP11 on KA cells disappeared. Three biological replicates of each treatment were performed.

We knocked down the expression of the BCR-ABL fusion protein in K562 and KA with lentivirus-packaged plasmids. As shown, the expression of P210 was significantly reduced, but the expression of SRC protein in shP210 K562 and shP210 KA cell lines did not decrease (Figure 4(a)); we used cell titer reagent to measure the cell viability of **DMP11** on the shP210K562 and shP210 KA. The dose-dependent inhibitory effect of **DMP11** on both cells disappeared (Figures 4(c) and 4(d)); however, when we preadded the proteasome inhibitor MG-132 before **DMP11**-incubated K562 and KA cell lines, the expression of fusion protein did not decrease relative to the control group after addition of the proteasome inhibitor, and the expression of SRC was also significantly higher than in the control group (Figure 4(b)).

### 3.5. DMP11 has Therapeutic Effect on Imatinib-Resistant KA-Induced Orthotopic Animal Model

**Figure 5:**
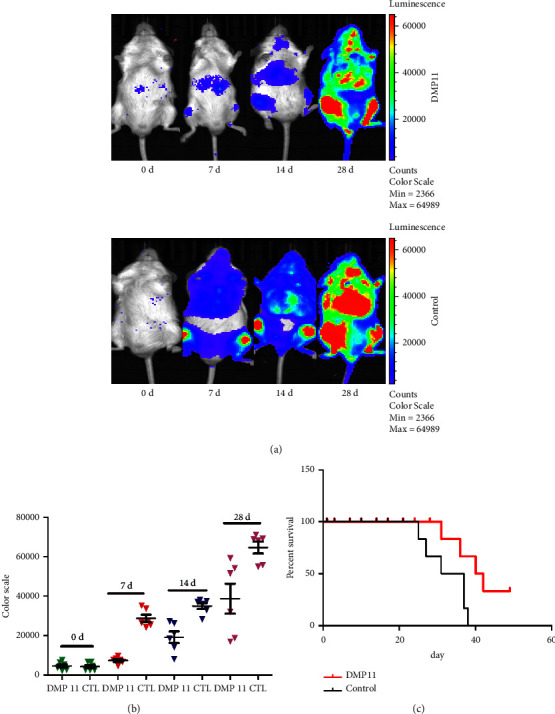
DMP11 has therapeutic effect on imatinib-resistant KA-induced orthotopic animal model. (a) Tumor progression in each group as analyzed by a small animal imaging system. (b) Comparison of fluorescence values between **DMP11** group and control group at different time points. (c) The effect of **DMP11** on the survival time of model mice. Three biological replicates of each treatment were performed and the data are presented as the mean ± SD. ^*∗*^*P* < 0.05; ^*∗∗*^*P* < 0.01; ^*∗∗∗*^*P* < 0.001; ^*∗∗∗∗*^*P* < 0.0001.

Biofluorescence was transfected into the KA cell line with lentivirus and then inoculated into NSG mice by tail vein injection. The results of small animal imaging showed that the fluorescent KA cells in the mice of the group administered with **DMP11** were obvious on the seventh day after inoculation. Compared with the control group, the fluorescence value was significantly reduced (Figures 5(a) and 5(b)); at the same time, compared with the control group, it was found that the survival period of the mice in the administration group was significantly prolonged. All mice died within 7 weeks, and 3 mice in the administration group survived (Figure 5(c)).

## 4. Discussion

The *Bcr-abl* fusion gene is translocated from chromosomes 9 and 22. The *Bcr-abl* fusion gene encodes a pathogenic protein (p210BCR-ABL) that continuously increases tyrosine kinase activity and activates multiple downstream signaling pathways to promote the occurrence and development of myeloid leukemia (chronic myeloid leukemia, CML) [25–27]. BCR-ABL kinase is closely related to SRC-family kinases (SFK), which make up the largest non-receptor tyrosine kinase family in the human body, including LYN, FYN, LCK, HCK, FGR, BLK, YRK, YES, and c-SRC [28–33]. They play a very important role in regulating cell proliferation, differentiation, adhesion, and movement [34–36], and abnormal expression of SFK members is related to the occurrence of various tumors in the human body [37–39]. Both BCR-ABL and SFK can promote the development of leukemia. Although BCR-ABL is traditionally believed to play a central role, SFK is also required for BCR-ABL to function, and they promote and depend on each other. More importantly, the interaction between BCR-ABL and SFK can induce imatinib resistance. This suggests that in targeted therapy for leukemia, SFK should be taken into account alongside BCR-ABL fusion protein. The interaction between BCR-ABL and SFK has a significant influence on tumor development [40–43]. We found that small molecule targeted degraders could simultaneously reduce BCR-ABL protein and SRC proteins in CML cell lines. At the same time, **DMP11** had a considerable inhibitory effect not only on wild-type CML cell lines but also on imatinib-resistant CML cell lines. As revealed by western blot analysis, DMP11 induced the degradation of BCR-ABL and SRC proteins in a time- and dose-dependent manner.

In our experiments, it was found that proteasome inhibitors can prevent the degradation of BCR-ABL protein by **DMP11**. The same situation was observed in imatinib-resistant CML cell lines, indicating that the degradation process is dependent on the ubiquitin-proteasome pathway. In wild-type, CML cell lines and imatinib-resistant CML cell lines, **DMP11** not only degraded the target protein BCR-ABL but also the SRC protein (Yes/Fyn/Fgr) in a time- and dose-dependent manner; when we preknocked down the fusion protein BCR-ABL, the dose-dependent inhibitory effect of **DMP11** on cells disappeared, but the degradation of SRC proteins (Yes/Fyn/Fgr) was not affected.

## 5. Conclusions

The ability of **DMP11** to overcome imatinib resistance is not achieved by degrading BCR-ABL and so indirectly degrades the SRC protein. It is more likely that **DMP11** achieves the dual-target degradation of BCR-ABL and SRC, and its good antiresistance effect in animal models was also very visible here. More in-depth research is still ongoing.

## 6. Chemistry

Synthetic route of the target compound: intermediates **5–8** were prepared according to our previous report.

## Data Availability

The data used to support the findings of this study are included within the article.

## Supplementary Materials

Supplementary Materials DMP6. ^1^H NMR (400 MHz, DMSO) *δ* 11.52 (s, 1H), 11.03 (s, 1H), 9.90 (s, 1H), 9.77 (s, 1H), 8.81 (s, 2H), 8.34 (t, *J* = 5.4 Hz, 1H), 8.24 (s, 1H), 7.82 (dd, *J* = 7.3, 1.4 Hz, 1H), 7.61-7.45 (m, 2H), 7.40 (dd, *J* = 7.3, 1.4 Hz, 1H), 7.32-7.13 (m, 2H), 6.12 (s, 1H), 5.16 (dd, *J* = 13.3, 5.1 Hz, 1H), 4.38 (q, *J* = 17.5 Hz, 2H), 3.93 (t, *J* = 5.3 Hz, 4H), 3.75-3.61 (m, 4H), 3.24 (dd, *J* = 12.6, 6.4 Hz, 2H), 2.99-2.83 (m, 1H), 2.68-2.56 (m, 1H), 2.45 (s, 3H), 2.40-2.30 (m, 3H), 2.25 (s, 3H), 2.03 (dd, *J* = 8.8, 3.7 Hz, 1H), 1.70 - 1.57 (m, 2H), 1.56-1.47 (m, 2H), 1.37-1.27 (m, 6H); ^13^C NMR (100 MHz, DMSO) *δ* 173.3, 171.9, 171.6, 168.3, 165.7, 163.9, 163.0, 162.7, 161.8, 160.4, 158.0, 157.4, 141.3, 139.3, 134.3, 134.2, 133.9, 133.1, 132.9, 129.5, 129.1, 128.6, 127.5, 126.2, 125.7, 119.5, 117.0, 83.2, 52.0, 46.9, 43.6, 43.3, 40.6, 40.3, 40.1, 36.2, 31.7, 29.6, 29.1, 29.0, 26.8, 26.1, 25.5, 23.1, 18.8. DMP7. ^1^H NMR (400 MHz, DMSO) *δ* 11.52 (s, 1H), 11.03 (s, 1H), 9.90 (s, 1H), 9.77 (s, 1H), 8.81 (s, 2H), 8.34 (t, *J* = 5.5 Hz, 1H), 8.24 (s, 1H), 7.82 (dd, *J* = 7.2, 1.6 Hz, 1H), 7.54-7.44 (m, 2H), 7.42-7.38 (m, 1H), 7.32-7.21 (m, 2H), 6.12 (s, 1H), 5.15 (dd, *J* = 13.3, 5.1 Hz, 1H), 4.37 (q, *J* = 17.5 Hz, 2H), 3.93 (d, *J* = 5.5 Hz, 4H), 3.72-3.63 (m, 4H), 3.25-3.16 (m, 2H), 3.00-2.84 (m, 1H), 2.65-2.57 (m, 1H), 2.44 (s, 3H), 2.40-2.32 (m, 3H), 2.25 (s, 3H), 2.09-1.98 (m, 1H), 1.64-1.56 (m, 2H), 1.53-1.46 (m, 2H), 1.29-1.21 (m, 18H); ^13^C NMR (100 MHz, DMSO) *δ* 173.3, 171.8, 171.5, 168.3, 165.6, 163.8, 163.0, 162.8, 161.8, 160.4, 158.0, 157.4, 141.3, 139.3, 134.3, 134.1, 134.0, 133.1, 129.3, 129.1, 127.5, 126.2, 125.7, 119.4, 117.0, 83.2, 52.0, 46.9, 43.5, 43.3, 36.3 31.6, 30.2, 29.6, 29.4, 29.2, 29.1, 26.9, 26.0, 25.6, 23.1, 22.6, 18.7, 14.4. DMP11.^1^H NMR (400 MHz, DMSO) *δ* 11.50 (s, 1H), 11.02 (s, 1H), 9.88 (s, 1H), 9.77 (s, 1H), 8.79 (s, 2H), 8.36 (t, *J* = 5.5 Hz, 1H), 8.24 (s, 1H), 7.81 (dd, *J* = 7.5, 1.2 Hz, 1H), 7.51 (dd, *J* = 7.5, 1.2 Hz, 1H), 7.49-7.44 (m, 1H), 7.39 (t, *J* = 7.4, 1.7 Hz, 1H), 7.30-7.26 (m, 1H), 7.27-7.21 (m, 1H), 6.12 (s, 1H), 5.15 (dd, *J* = 13.3, 5.1 Hz, 1H), 4.47-4.26 (m, 2H), 4.03-3.87 (m, 4H), 3.73-3.63 (m, 4H), 3.28-3.22 (m, 2H), 3.09 (qd, *J* = 7.3, 4.8 Hz, 1H), 2.98-2.82 (m, 1H), 2.67-2.57 (m, 1H), 2.45 (s, 3H), 2.41-2.33 (mz, 2H), 2.26 (s, 3H), 2.08-2.00 (m, 1H), 1.71-1.62 (m, 2H), 1.61-1.53 (m, 2H), 1.45-1.35 (m, 2H), 1.28-1.15 (m, 4H); ^13^C NMR (100 MHz, DMSO) *δ* 173.2, 171.8, 171.5, 168.3, 165.6, 163.9, 163.1, 162.7, 161.8, 160.4, 157.9, 157.5, 141.3, 139.3, 134.2, 134.1, 134.0, 133.1, 132.9, 129.4, 128.9, 128.5, 127.4, 126.2, 125.7, 119.4, 117.0, 83.3, 79.7, 79.4, 79.1, 55.6, 52.0, 46.9, 46.1, 43.6, 43.3, 36.2, 31.7, 29.4, 26.5, 26.0, 25.3, 23.1, 18.8, 9.0. DMP12.^1^H NMR (400 MHz, DMSO) *δ* 11.51 (s, 1H), 11.03 (s, 1H), 9.90 (s, 1H), 9.78 (s, 1H), 8.81 (s, 2H), 8.35 (t, *J* = 5.5 Hz, 1H), 8.25 (s, 1H), 7.82 (dd, *J* = 7.2, 1.6 Hz, 1H), 7.53-7.47 (m, 2H), 7.40 (dd, *J* = 7.2, 1.6 Hz, 1H), 7.31-7.25 (m, 2H), 6.12 (s, 2H), 5.16 (dd, *J* = 13.3, 5.1 Hz, 1H), 4.39-4.35 (m, 2H), 3.93 (d, *J* = 5.5 Hz, 6H), 3.70-3.64 (m, 4H), 3.25-3.11 (m, 2H), 3.09-2.82 (m, 2H), 2.70-2.54 (m, 2H), 2.45 (s, 3H), 2.36-2.34 (m, 3H), 2.25 (s, 3H), 2.08-1.95 (m, 1H), 1.70-1.57 (m, 2H), 1.50-1.47 (m, 4H), 1.36-1.21 (m, 14H); ^13^C NMR (101 MHz, DMSO) *δ* 173.3, 171.8, 171.5, 168.3, 165.6, 163.8, 163.0, 162.7, 161.8, 160.4, 158.0, 157.4, 141.3, 139.3, 134.3, 134.1, 134.0, 133.1, 132.9, 129.5, 129.1, 128.6, 127.4, 126.2, 125.7, 123.2, 119.4, 117.0, 116.0, 107.4, 97.6, 83.2, 68.7, 67.1, 52.0, 46.9, 46.1, 43.6, 43.3, 36.2, 34.8, 32.0, 31.6, 29.9, 29.6, 29.5, 29.4, 29.2, 29.1, 27.8, 26.9, 26.0, 25.5, 24.0, 23.1, 22.2, 18.7, 9.3.
