# Supplementary figures and images for: The Activity of Novel BCR-ABL Small-Molecule Degraders Containing Pyrimidine Rings and Their Role in Overcoming Drug Resistance
Source: J Oncol. 2022 Oct 30;2022:4056398. doi: 10.1155/2022/4056398 (PMC9637472; doi:10.1155/2022/4056398)

z.sx-011

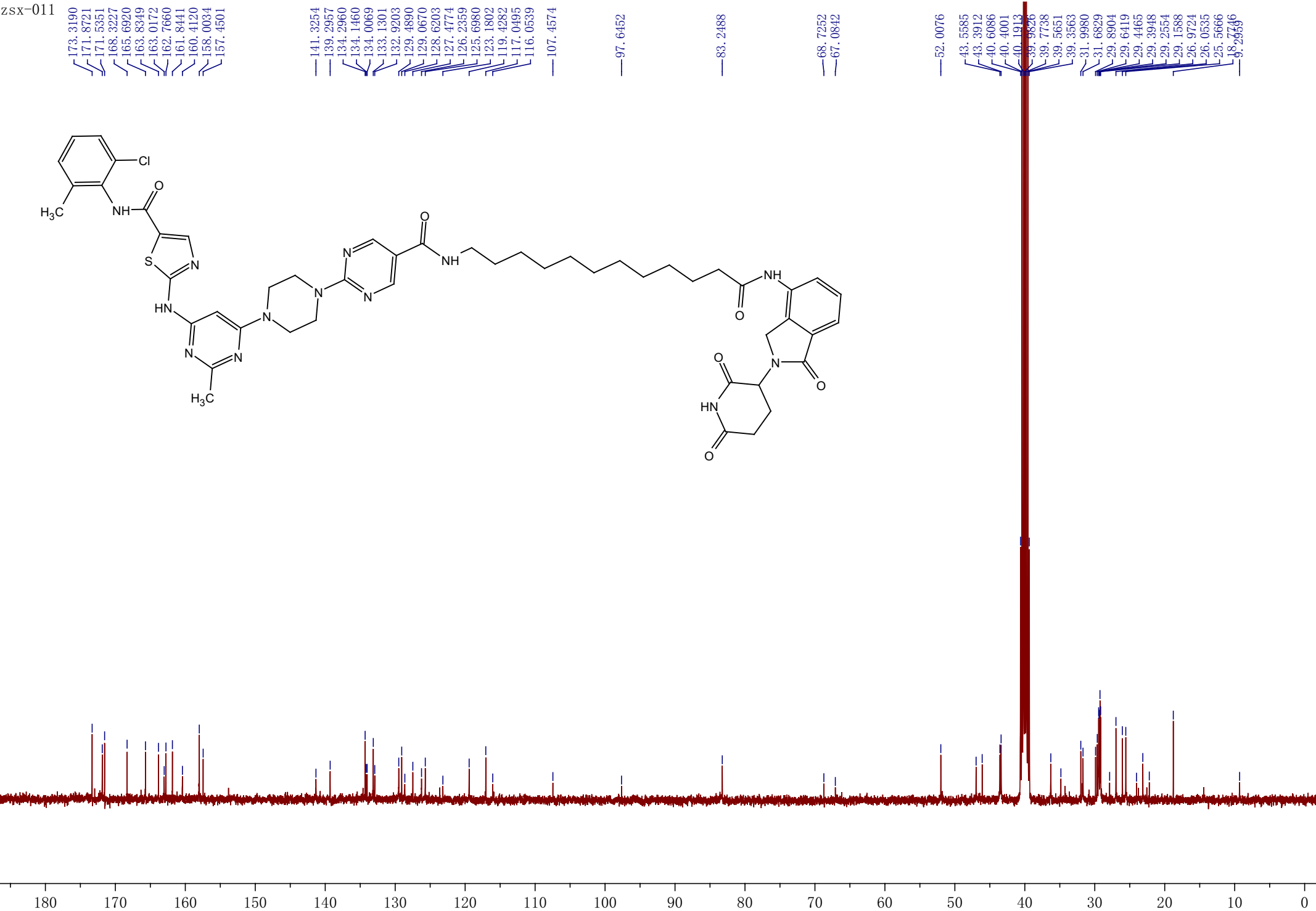

Supplement: Supplementary Materials — DMP6. 1H NMR (400 MHz, DMSO) δ 11.52 (s, 1H), 11.03 (s, 1H), 9.90 (s, 1H), 9.77 (s, 1H), 8.81 (s, 2H), 8.34 (t, J = 5.4 Hz, 1H), 8.24 (s, 1H), 7.82 (dd, J = 7.3, 1.4 Hz, 1H), 7.61-7.45 (m, 2H), 7.40 (dd, J = 7.3, 1.4 Hz, 1H), 7.32-7.13 (m, 2H), 6.12 (s, 1H), 5.16 (dd, J = 13.3, 5.1 Hz, 1H), 4.38 (q, J = 17.5 Hz, 2H), 3.93 (t, J = 5.3 Hz, 4H), 3.75-3.61 (m, 4H), 3.24 (dd, J = 12.6, 6.4 Hz, 2H), 2.99-2.83 (m, 1H), 2.68-2.56 (m, 1H), 2.45 (s, 3H), 2.40-2.30 (m, 3H), 2.25 (s, 3H), 2.03 (dd, J = 8.8, 3.7 Hz, 1H), 1.70 - 1.57 (m, 2H), 1.56-1.47 (m, 2H), 1.37-1.27 (m, 6H); 13C NMR (100 MHz, DMSO) δ 173.3, 171.9, 171.6, 168.3, 165.7, 163.9, 163.0, 162.7, 161.8, 160.4, 158.0, 157.4, 141.3, 139.3, 134.3, 134.2, 133.9, 133.1, 132.9, 129.5, 129.1, 128.6, 127.5, 126.2, 125.7, 119.5, 117.0, 83.2, 52.0, 46.9, 43.6, 43.3, 40.6, 40.3, 40.1, 36.2, 31.7, 29.6, 29.1, 29.0, 26.8, 26.1, 25.5, 23.1, 18.8. DMP7. 1H NMR (400 MHz, DMSO) δ 11.52 (s, 1H), 11.03 (s, 1H), 9.90 (s, 1H), 9.77 (s, 1H), 8.81 (s, 2H), 8.34 (t, J = 5.5 Hz, 1H), 8.24 (s, 1H), 7.82 (dd, J = 7.2, 1.6 Hz, 1H), 7.54-7.44 (m, 2H), 7.42-7.38 (m, 1H), 7.32-7.21 (m, 2H), 6.12 (s, 1H), 5.15 (dd, J = 13.3, 5.1 Hz, 1H), 4.37 (q, J = 17.5 Hz, 2H), 3.93 (d, J = 5.5 Hz, 4H), 3.72-3.63 (m, 4H), 3.25-3.16 (m, 2H), 3.00-2.84 (m, 1H), 2.65-2.57 (m, 1H), 2.44 (s, 3H), 2.40-2.32 (m, 3H), 2.25 (s, 3H), 2.09-1.98 (m, 1H), 1.64-1.56 (m, 2H), 1.53-1.46 (m, 2H), 1.29-1.21 (m, 18H); 13C NMR (100 MHz, DMSO) δ 173.3, 171.8, 171.5, 168.3, 165.6, 163.8, 163.0, 162.8, 161.8, 160.4, 158.0, 157.4, 141.3, 139.3, 134.3, 134.1, 134.0, 133.1, 129.3, 129.1, 127.5, 126.2, 125.7, 119.4, 117.0, 83.2, 52.0, 46.9, 43.5, 43.3, 36.3 31.6, 30.2, 29.6, 29.4, 29.2, 29.1, 26.9, 26.0, 25.6, 23.1, 22.6, 18.7, 14.4. DMP11.1H NMR (400 MHz, DMSO) δ 11.50 (s, 1H), 11.02 (s, 1H), 9.88 (s, 1H), 9.77 (s, 1H), 8.79 (s, 2H), 8.36 (t, J = 5.5 Hz, 1H), 8.24 (s, 1H), 7.81 (dd, J = 7.5, 1.2 Hz, 1H), 7.51 (dd, J = 7.5, 1.2 Hz, 1H), 7.49-7.44 (m, 1H), 7.39 (t, J = 7.4, 1.7 Hz, 1H), 7 [file 4056398.f1.zip › DMP-11-C NMR.pdf]

z.sx-011

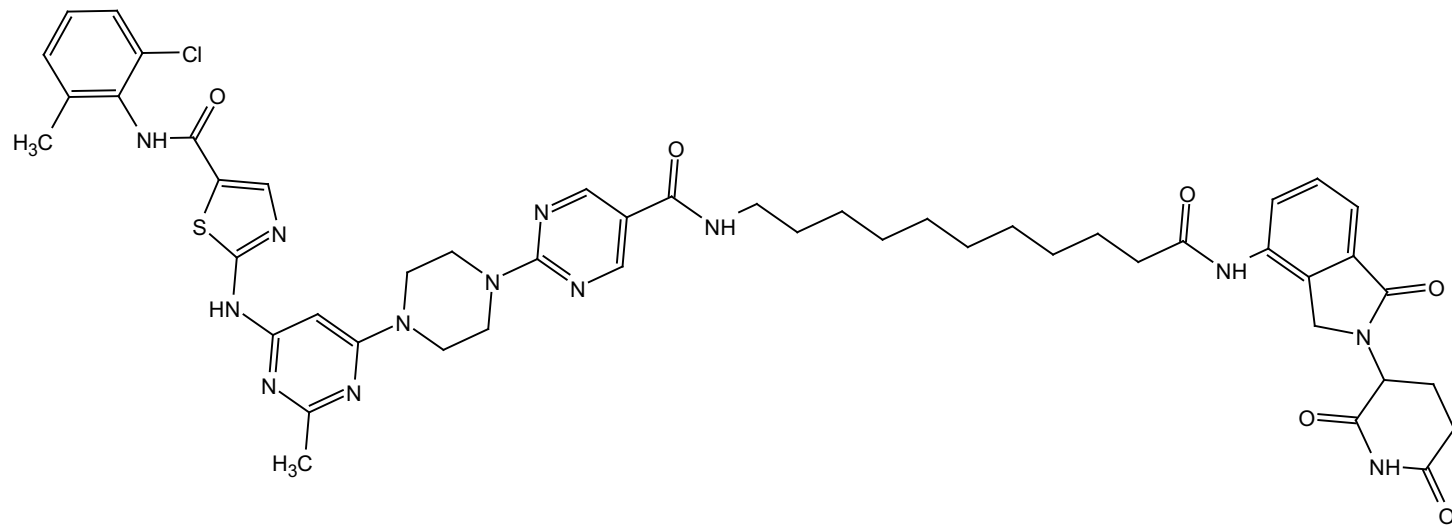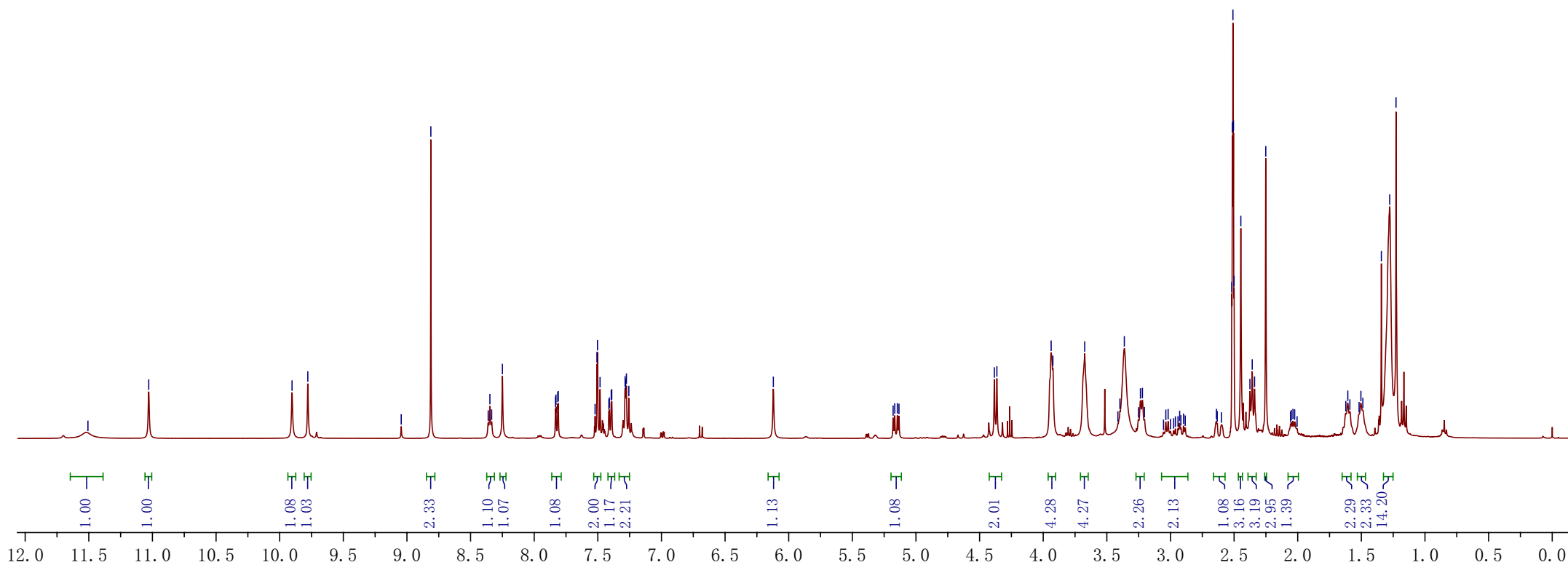

Supplement: Supplementary Materials — DMP6. 1H NMR (400 MHz, DMSO) δ 11.52 (s, 1H), 11.03 (s, 1H), 9.90 (s, 1H), 9.77 (s, 1H), 8.81 (s, 2H), 8.34 (t, J = 5.4 Hz, 1H), 8.24 (s, 1H), 7.82 (dd, J = 7.3, 1.4 Hz, 1H), 7.61-7.45 (m, 2H), 7.40 (dd, J = 7.3, 1.4 Hz, 1H), 7.32-7.13 (m, 2H), 6.12 (s, 1H), 5.16 (dd, J = 13.3, 5.1 Hz, 1H), 4.38 (q, J = 17.5 Hz, 2H), 3.93 (t, J = 5.3 Hz, 4H), 3.75-3.61 (m, 4H), 3.24 (dd, J = 12.6, 6.4 Hz, 2H), 2.99-2.83 (m, 1H), 2.68-2.56 (m, 1H), 2.45 (s, 3H), 2.40-2.30 (m, 3H), 2.25 (s, 3H), 2.03 (dd, J = 8.8, 3.7 Hz, 1H), 1.70 - 1.57 (m, 2H), 1.56-1.47 (m, 2H), 1.37-1.27 (m, 6H); 13C NMR (100 MHz, DMSO) δ 173.3, 171.9, 171.6, 168.3, 165.7, 163.9, 163.0, 162.7, 161.8, 160.4, 158.0, 157.4, 141.3, 139.3, 134.3, 134.2, 133.9, 133.1, 132.9, 129.5, 129.1, 128.6, 127.5, 126.2, 125.7, 119.5, 117.0, 83.2, 52.0, 46.9, 43.6, 43.3, 40.6, 40.3, 40.1, 36.2, 31.7, 29.6, 29.1, 29.0, 26.8, 26.1, 25.5, 23.1, 18.8. DMP7. 1H NMR (400 MHz, DMSO) δ 11.52 (s, 1H), 11.03 (s, 1H), 9.90 (s, 1H), 9.77 (s, 1H), 8.81 (s, 2H), 8.34 (t, J = 5.5 Hz, 1H), 8.24 (s, 1H), 7.82 (dd, J = 7.2, 1.6 Hz, 1H), 7.54-7.44 (m, 2H), 7.42-7.38 (m, 1H), 7.32-7.21 (m, 2H), 6.12 (s, 1H), 5.15 (dd, J = 13.3, 5.1 Hz, 1H), 4.37 (q, J = 17.5 Hz, 2H), 3.93 (d, J = 5.5 Hz, 4H), 3.72-3.63 (m, 4H), 3.25-3.16 (m, 2H), 3.00-2.84 (m, 1H), 2.65-2.57 (m, 1H), 2.44 (s, 3H), 2.40-2.32 (m, 3H), 2.25 (s, 3H), 2.09-1.98 (m, 1H), 1.64-1.56 (m, 2H), 1.53-1.46 (m, 2H), 1.29-1.21 (m, 18H); 13C NMR (100 MHz, DMSO) δ 173.3, 171.8, 171.5, 168.3, 165.6, 163.8, 163.0, 162.8, 161.8, 160.4, 158.0, 157.4, 141.3, 139.3, 134.3, 134.1, 134.0, 133.1, 129.3, 129.1, 127.5, 126.2, 125.7, 119.4, 117.0, 83.2, 52.0, 46.9, 43.5, 43.3, 36.3 31.6, 30.2, 29.6, 29.4, 29.2, 29.1, 26.9, 26.0, 25.6, 23.1, 22.6, 18.7, 14.4. DMP11.1H NMR (400 MHz, DMSO) δ 11.50 (s, 1H), 11.02 (s, 1H), 9.88 (s, 1H), 9.77 (s, 1H), 8.79 (s, 2H), 8.36 (t, J = 5.5 Hz, 1H), 8.24 (s, 1H), 7.81 (dd, J = 7.5, 1.2 Hz, 1H), 7.51 (dd, J = 7.5, 1.2 Hz, 1H), 7.49-7.44 (m, 1H), 7.39 (t, J = 7.4, 1.7 Hz, 1H), 7 [file 4056398.f1.zip › DMP-11-H NMR.pdf]

zsx-012

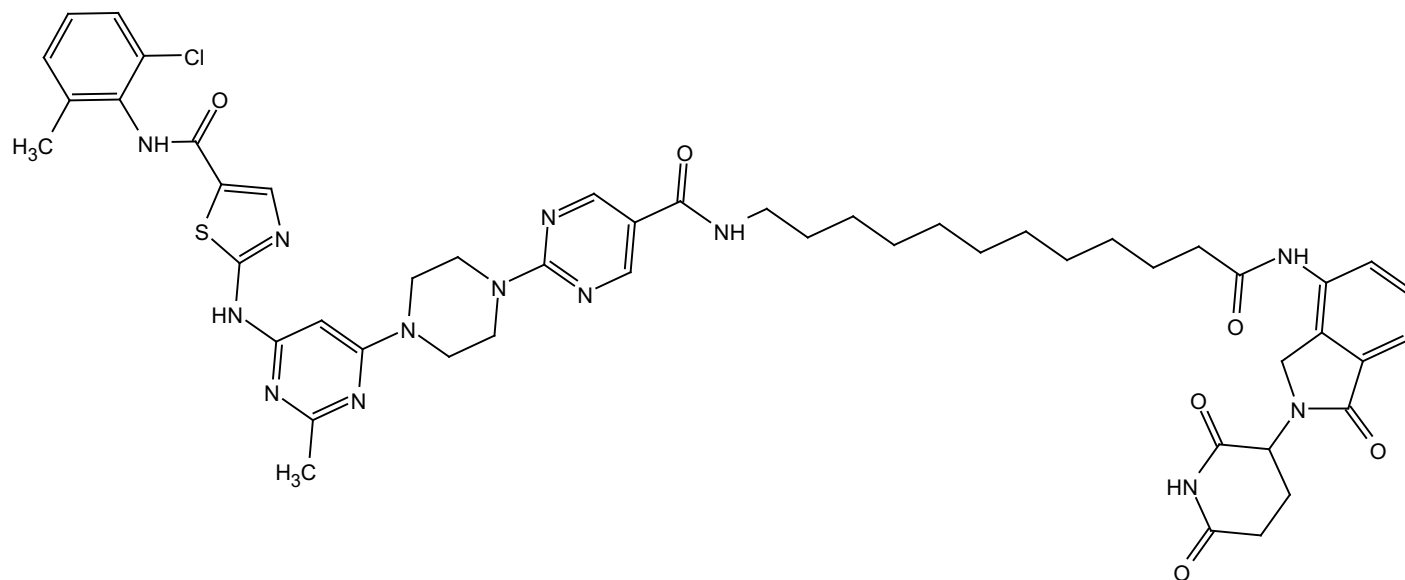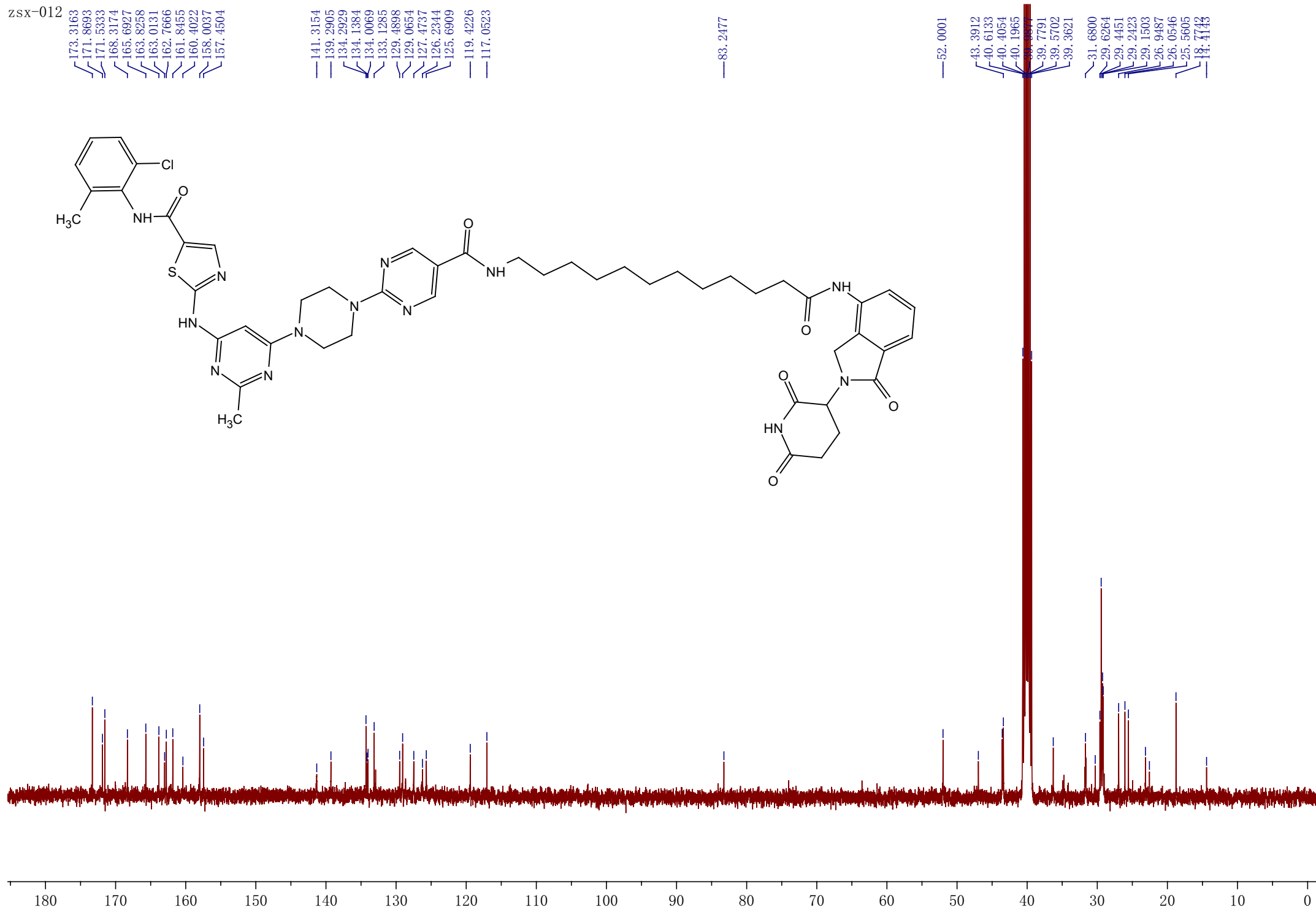

Supplement: Supplementary Materials — DMP6. 1H NMR (400 MHz, DMSO) δ 11.52 (s, 1H), 11.03 (s, 1H), 9.90 (s, 1H), 9.77 (s, 1H), 8.81 (s, 2H), 8.34 (t, J = 5.4 Hz, 1H), 8.24 (s, 1H), 7.82 (dd, J = 7.3, 1.4 Hz, 1H), 7.61-7.45 (m, 2H), 7.40 (dd, J = 7.3, 1.4 Hz, 1H), 7.32-7.13 (m, 2H), 6.12 (s, 1H), 5.16 (dd, J = 13.3, 5.1 Hz, 1H), 4.38 (q, J = 17.5 Hz, 2H), 3.93 (t, J = 5.3 Hz, 4H), 3.75-3.61 (m, 4H), 3.24 (dd, J = 12.6, 6.4 Hz, 2H), 2.99-2.83 (m, 1H), 2.68-2.56 (m, 1H), 2.45 (s, 3H), 2.40-2.30 (m, 3H), 2.25 (s, 3H), 2.03 (dd, J = 8.8, 3.7 Hz, 1H), 1.70 - 1.57 (m, 2H), 1.56-1.47 (m, 2H), 1.37-1.27 (m, 6H); 13C NMR (100 MHz, DMSO) δ 173.3, 171.9, 171.6, 168.3, 165.7, 163.9, 163.0, 162.7, 161.8, 160.4, 158.0, 157.4, 141.3, 139.3, 134.3, 134.2, 133.9, 133.1, 132.9, 129.5, 129.1, 128.6, 127.5, 126.2, 125.7, 119.5, 117.0, 83.2, 52.0, 46.9, 43.6, 43.3, 40.6, 40.3, 40.1, 36.2, 31.7, 29.6, 29.1, 29.0, 26.8, 26.1, 25.5, 23.1, 18.8. DMP7. 1H NMR (400 MHz, DMSO) δ 11.52 (s, 1H), 11.03 (s, 1H), 9.90 (s, 1H), 9.77 (s, 1H), 8.81 (s, 2H), 8.34 (t, J = 5.5 Hz, 1H), 8.24 (s, 1H), 7.82 (dd, J = 7.2, 1.6 Hz, 1H), 7.54-7.44 (m, 2H), 7.42-7.38 (m, 1H), 7.32-7.21 (m, 2H), 6.12 (s, 1H), 5.15 (dd, J = 13.3, 5.1 Hz, 1H), 4.37 (q, J = 17.5 Hz, 2H), 3.93 (d, J = 5.5 Hz, 4H), 3.72-3.63 (m, 4H), 3.25-3.16 (m, 2H), 3.00-2.84 (m, 1H), 2.65-2.57 (m, 1H), 2.44 (s, 3H), 2.40-2.32 (m, 3H), 2.25 (s, 3H), 2.09-1.98 (m, 1H), 1.64-1.56 (m, 2H), 1.53-1.46 (m, 2H), 1.29-1.21 (m, 18H); 13C NMR (100 MHz, DMSO) δ 173.3, 171.8, 171.5, 168.3, 165.6, 163.8, 163.0, 162.8, 161.8, 160.4, 158.0, 157.4, 141.3, 139.3, 134.3, 134.1, 134.0, 133.1, 129.3, 129.1, 127.5, 126.2, 125.7, 119.4, 117.0, 83.2, 52.0, 46.9, 43.5, 43.3, 36.3 31.6, 30.2, 29.6, 29.4, 29.2, 29.1, 26.9, 26.0, 25.6, 23.1, 22.6, 18.7, 14.4. DMP11.1H NMR (400 MHz, DMSO) δ 11.50 (s, 1H), 11.02 (s, 1H), 9.88 (s, 1H), 9.77 (s, 1H), 8.79 (s, 2H), 8.36 (t, J = 5.5 Hz, 1H), 8.24 (s, 1H), 7.81 (dd, J = 7.5, 1.2 Hz, 1H), 7.51 (dd, J = 7.5, 1.2 Hz, 1H), 7.49-7.44 (m, 1H), 7.39 (t, J = 7.4, 1.7 Hz, 1H), 7 [file 4056398.f1.zip › DMP-12-C NMR.pdf]

z.sx-012

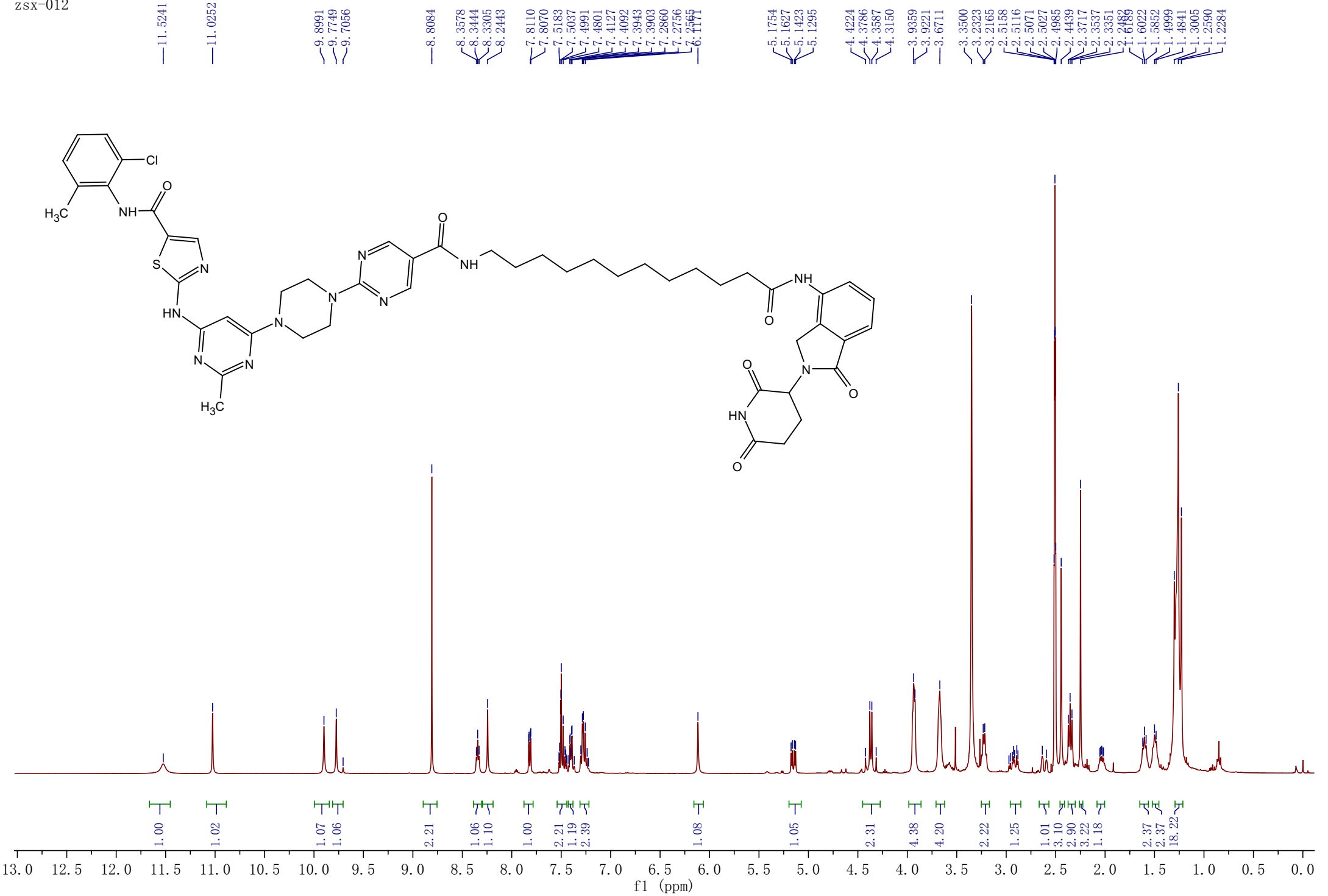

Supplement: Supplementary Materials — DMP6. 1H NMR (400 MHz, DMSO) δ 11.52 (s, 1H), 11.03 (s, 1H), 9.90 (s, 1H), 9.77 (s, 1H), 8.81 (s, 2H), 8.34 (t, J = 5.4 Hz, 1H), 8.24 (s, 1H), 7.82 (dd, J = 7.3, 1.4 Hz, 1H), 7.61-7.45 (m, 2H), 7.40 (dd, J = 7.3, 1.4 Hz, 1H), 7.32-7.13 (m, 2H), 6.12 (s, 1H), 5.16 (dd, J = 13.3, 5.1 Hz, 1H), 4.38 (q, J = 17.5 Hz, 2H), 3.93 (t, J = 5.3 Hz, 4H), 3.75-3.61 (m, 4H), 3.24 (dd, J = 12.6, 6.4 Hz, 2H), 2.99-2.83 (m, 1H), 2.68-2.56 (m, 1H), 2.45 (s, 3H), 2.40-2.30 (m, 3H), 2.25 (s, 3H), 2.03 (dd, J = 8.8, 3.7 Hz, 1H), 1.70 - 1.57 (m, 2H), 1.56-1.47 (m, 2H), 1.37-1.27 (m, 6H); 13C NMR (100 MHz, DMSO) δ 173.3, 171.9, 171.6, 168.3, 165.7, 163.9, 163.0, 162.7, 161.8, 160.4, 158.0, 157.4, 141.3, 139.3, 134.3, 134.2, 133.9, 133.1, 132.9, 129.5, 129.1, 128.6, 127.5, 126.2, 125.7, 119.5, 117.0, 83.2, 52.0, 46.9, 43.6, 43.3, 40.6, 40.3, 40.1, 36.2, 31.7, 29.6, 29.1, 29.0, 26.8, 26.1, 25.5, 23.1, 18.8. DMP7. 1H NMR (400 MHz, DMSO) δ 11.52 (s, 1H), 11.03 (s, 1H), 9.90 (s, 1H), 9.77 (s, 1H), 8.81 (s, 2H), 8.34 (t, J = 5.5 Hz, 1H), 8.24 (s, 1H), 7.82 (dd, J = 7.2, 1.6 Hz, 1H), 7.54-7.44 (m, 2H), 7.42-7.38 (m, 1H), 7.32-7.21 (m, 2H), 6.12 (s, 1H), 5.15 (dd, J = 13.3, 5.1 Hz, 1H), 4.37 (q, J = 17.5 Hz, 2H), 3.93 (d, J = 5.5 Hz, 4H), 3.72-3.63 (m, 4H), 3.25-3.16 (m, 2H), 3.00-2.84 (m, 1H), 2.65-2.57 (m, 1H), 2.44 (s, 3H), 2.40-2.32 (m, 3H), 2.25 (s, 3H), 2.09-1.98 (m, 1H), 1.64-1.56 (m, 2H), 1.53-1.46 (m, 2H), 1.29-1.21 (m, 18H); 13C NMR (100 MHz, DMSO) δ 173.3, 171.8, 171.5, 168.3, 165.6, 163.8, 163.0, 162.8, 161.8, 160.4, 158.0, 157.4, 141.3, 139.3, 134.3, 134.1, 134.0, 133.1, 129.3, 129.1, 127.5, 126.2, 125.7, 119.4, 117.0, 83.2, 52.0, 46.9, 43.5, 43.3, 36.3 31.6, 30.2, 29.6, 29.4, 29.2, 29.1, 26.9, 26.0, 25.6, 23.1, 22.6, 18.7, 14.4. DMP11.1H NMR (400 MHz, DMSO) δ 11.50 (s, 1H), 11.02 (s, 1H), 9.88 (s, 1H), 9.77 (s, 1H), 8.79 (s, 2H), 8.36 (t, J = 5.5 Hz, 1H), 8.24 (s, 1H), 7.81 (dd, J = 7.5, 1.2 Hz, 1H), 7.51 (dd, J = 7.5, 1.2 Hz, 1H), 7.49-7.44 (m, 1H), 7.39 (t, J = 7.4, 1.7 Hz, 1H), 7 [file 4056398.f1.zip › DMP-12-H NMR.pdf]

z.sx-006

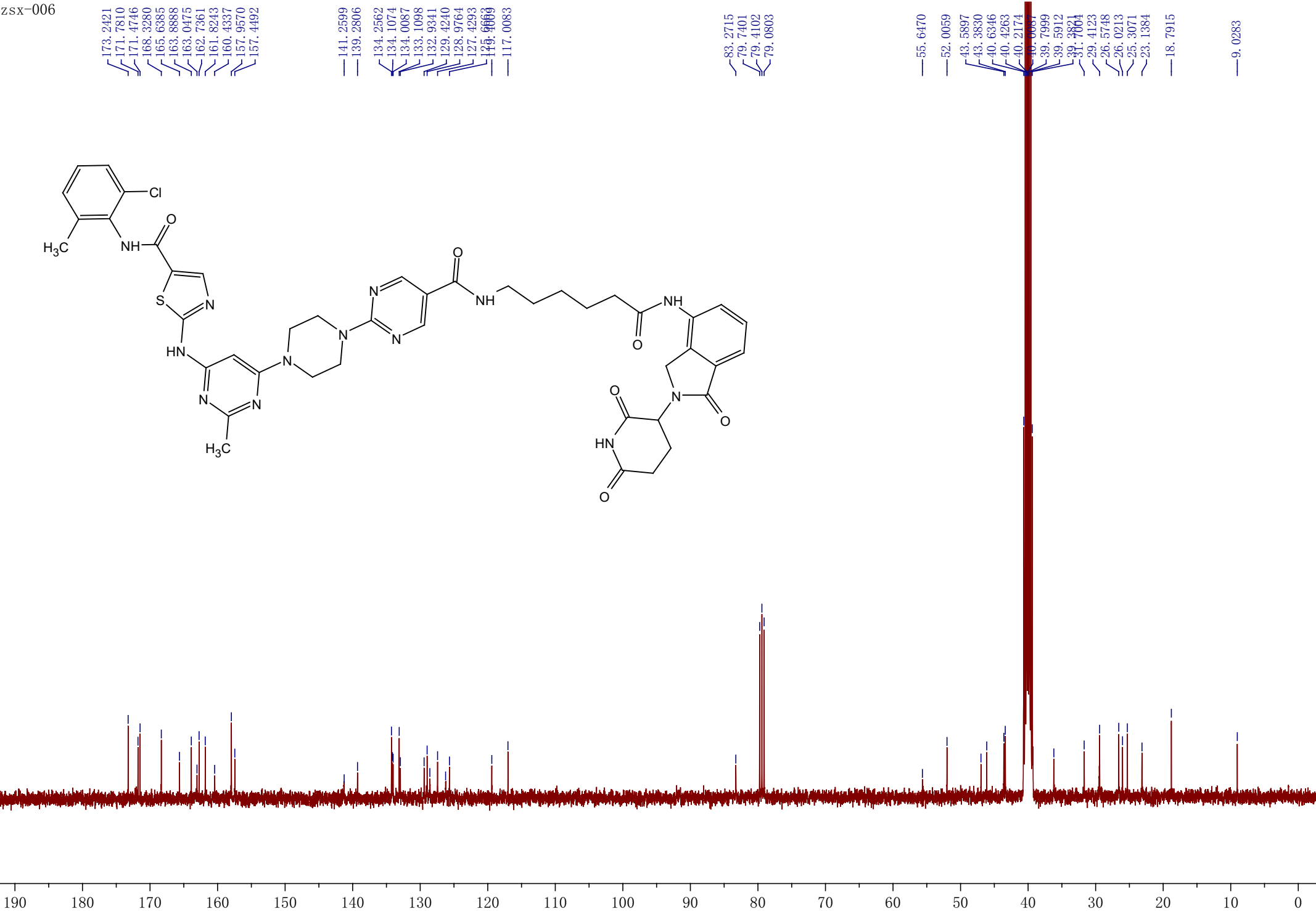

Supplement: Supplementary Materials — DMP6. 1H NMR (400 MHz, DMSO) δ 11.52 (s, 1H), 11.03 (s, 1H), 9.90 (s, 1H), 9.77 (s, 1H), 8.81 (s, 2H), 8.34 (t, J = 5.4 Hz, 1H), 8.24 (s, 1H), 7.82 (dd, J = 7.3, 1.4 Hz, 1H), 7.61-7.45 (m, 2H), 7.40 (dd, J = 7.3, 1.4 Hz, 1H), 7.32-7.13 (m, 2H), 6.12 (s, 1H), 5.16 (dd, J = 13.3, 5.1 Hz, 1H), 4.38 (q, J = 17.5 Hz, 2H), 3.93 (t, J = 5.3 Hz, 4H), 3.75-3.61 (m, 4H), 3.24 (dd, J = 12.6, 6.4 Hz, 2H), 2.99-2.83 (m, 1H), 2.68-2.56 (m, 1H), 2.45 (s, 3H), 2.40-2.30 (m, 3H), 2.25 (s, 3H), 2.03 (dd, J = 8.8, 3.7 Hz, 1H), 1.70 - 1.57 (m, 2H), 1.56-1.47 (m, 2H), 1.37-1.27 (m, 6H); 13C NMR (100 MHz, DMSO) δ 173.3, 171.9, 171.6, 168.3, 165.7, 163.9, 163.0, 162.7, 161.8, 160.4, 158.0, 157.4, 141.3, 139.3, 134.3, 134.2, 133.9, 133.1, 132.9, 129.5, 129.1, 128.6, 127.5, 126.2, 125.7, 119.5, 117.0, 83.2, 52.0, 46.9, 43.6, 43.3, 40.6, 40.3, 40.1, 36.2, 31.7, 29.6, 29.1, 29.0, 26.8, 26.1, 25.5, 23.1, 18.8. DMP7. 1H NMR (400 MHz, DMSO) δ 11.52 (s, 1H), 11.03 (s, 1H), 9.90 (s, 1H), 9.77 (s, 1H), 8.81 (s, 2H), 8.34 (t, J = 5.5 Hz, 1H), 8.24 (s, 1H), 7.82 (dd, J = 7.2, 1.6 Hz, 1H), 7.54-7.44 (m, 2H), 7.42-7.38 (m, 1H), 7.32-7.21 (m, 2H), 6.12 (s, 1H), 5.15 (dd, J = 13.3, 5.1 Hz, 1H), 4.37 (q, J = 17.5 Hz, 2H), 3.93 (d, J = 5.5 Hz, 4H), 3.72-3.63 (m, 4H), 3.25-3.16 (m, 2H), 3.00-2.84 (m, 1H), 2.65-2.57 (m, 1H), 2.44 (s, 3H), 2.40-2.32 (m, 3H), 2.25 (s, 3H), 2.09-1.98 (m, 1H), 1.64-1.56 (m, 2H), 1.53-1.46 (m, 2H), 1.29-1.21 (m, 18H); 13C NMR (100 MHz, DMSO) δ 173.3, 171.8, 171.5, 168.3, 165.6, 163.8, 163.0, 162.8, 161.8, 160.4, 158.0, 157.4, 141.3, 139.3, 134.3, 134.1, 134.0, 133.1, 129.3, 129.1, 127.5, 126.2, 125.7, 119.4, 117.0, 83.2, 52.0, 46.9, 43.5, 43.3, 36.3 31.6, 30.2, 29.6, 29.4, 29.2, 29.1, 26.9, 26.0, 25.6, 23.1, 22.6, 18.7, 14.4. DMP11.1H NMR (400 MHz, DMSO) δ 11.50 (s, 1H), 11.02 (s, 1H), 9.88 (s, 1H), 9.77 (s, 1H), 8.79 (s, 2H), 8.36 (t, J = 5.5 Hz, 1H), 8.24 (s, 1H), 7.81 (dd, J = 7.5, 1.2 Hz, 1H), 7.51 (dd, J = 7.5, 1.2 Hz, 1H), 7.49-7.44 (m, 1H), 7.39 (t, J = 7.4, 1.7 Hz, 1H), 7 [file 4056398.f1.zip › DMP-6-C NMR.pdf]

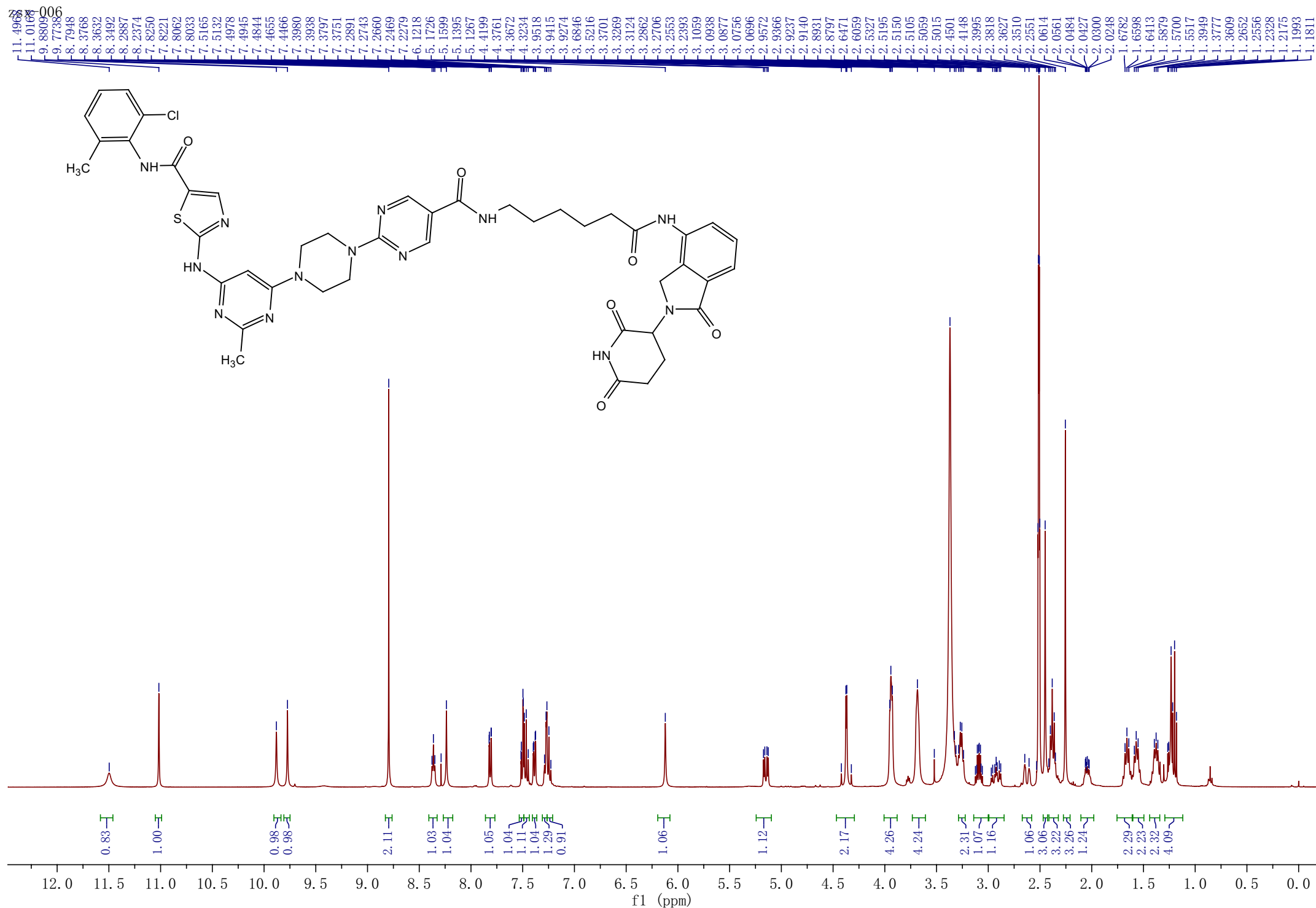

Supplement: Supplementary Materials — DMP6. 1H NMR (400 MHz, DMSO) δ 11.52 (s, 1H), 11.03 (s, 1H), 9.90 (s, 1H), 9.77 (s, 1H), 8.81 (s, 2H), 8.34 (t, J = 5.4 Hz, 1H), 8.24 (s, 1H), 7.82 (dd, J = 7.3, 1.4 Hz, 1H), 7.61-7.45 (m, 2H), 7.40 (dd, J = 7.3, 1.4 Hz, 1H), 7.32-7.13 (m, 2H), 6.12 (s, 1H), 5.16 (dd, J = 13.3, 5.1 Hz, 1H), 4.38 (q, J = 17.5 Hz, 2H), 3.93 (t, J = 5.3 Hz, 4H), 3.75-3.61 (m, 4H), 3.24 (dd, J = 12.6, 6.4 Hz, 2H), 2.99-2.83 (m, 1H), 2.68-2.56 (m, 1H), 2.45 (s, 3H), 2.40-2.30 (m, 3H), 2.25 (s, 3H), 2.03 (dd, J = 8.8, 3.7 Hz, 1H), 1.70 - 1.57 (m, 2H), 1.56-1.47 (m, 2H), 1.37-1.27 (m, 6H); 13C NMR (100 MHz, DMSO) δ 173.3, 171.9, 171.6, 168.3, 165.7, 163.9, 163.0, 162.7, 161.8, 160.4, 158.0, 157.4, 141.3, 139.3, 134.3, 134.2, 133.9, 133.1, 132.9, 129.5, 129.1, 128.6, 127.5, 126.2, 125.7, 119.5, 117.0, 83.2, 52.0, 46.9, 43.6, 43.3, 40.6, 40.3, 40.1, 36.2, 31.7, 29.6, 29.1, 29.0, 26.8, 26.1, 25.5, 23.1, 18.8. DMP7. 1H NMR (400 MHz, DMSO) δ 11.52 (s, 1H), 11.03 (s, 1H), 9.90 (s, 1H), 9.77 (s, 1H), 8.81 (s, 2H), 8.34 (t, J = 5.5 Hz, 1H), 8.24 (s, 1H), 7.82 (dd, J = 7.2, 1.6 Hz, 1H), 7.54-7.44 (m, 2H), 7.42-7.38 (m, 1H), 7.32-7.21 (m, 2H), 6.12 (s, 1H), 5.15 (dd, J = 13.3, 5.1 Hz, 1H), 4.37 (q, J = 17.5 Hz, 2H), 3.93 (d, J = 5.5 Hz, 4H), 3.72-3.63 (m, 4H), 3.25-3.16 (m, 2H), 3.00-2.84 (m, 1H), 2.65-2.57 (m, 1H), 2.44 (s, 3H), 2.40-2.32 (m, 3H), 2.25 (s, 3H), 2.09-1.98 (m, 1H), 1.64-1.56 (m, 2H), 1.53-1.46 (m, 2H), 1.29-1.21 (m, 18H); 13C NMR (100 MHz, DMSO) δ 173.3, 171.8, 171.5, 168.3, 165.6, 163.8, 163.0, 162.8, 161.8, 160.4, 158.0, 157.4, 141.3, 139.3, 134.3, 134.1, 134.0, 133.1, 129.3, 129.1, 127.5, 126.2, 125.7, 119.4, 117.0, 83.2, 52.0, 46.9, 43.5, 43.3, 36.3 31.6, 30.2, 29.6, 29.4, 29.2, 29.1, 26.9, 26.0, 25.6, 23.1, 22.6, 18.7, 14.4. DMP11.1H NMR (400 MHz, DMSO) δ 11.50 (s, 1H), 11.02 (s, 1H), 9.88 (s, 1H), 9.77 (s, 1H), 8.79 (s, 2H), 8.36 (t, J = 5.5 Hz, 1H), 8.24 (s, 1H), 7.81 (dd, J = 7.5, 1.2 Hz, 1H), 7.51 (dd, J = 7.5, 1.2 Hz, 1H), 7.49-7.44 (m, 1H), 7.39 (t, J = 7.4, 1.7 Hz, 1H), 7 [file 4056398.f1.zip › DMP-6-H NMR.pdf]

zsx008

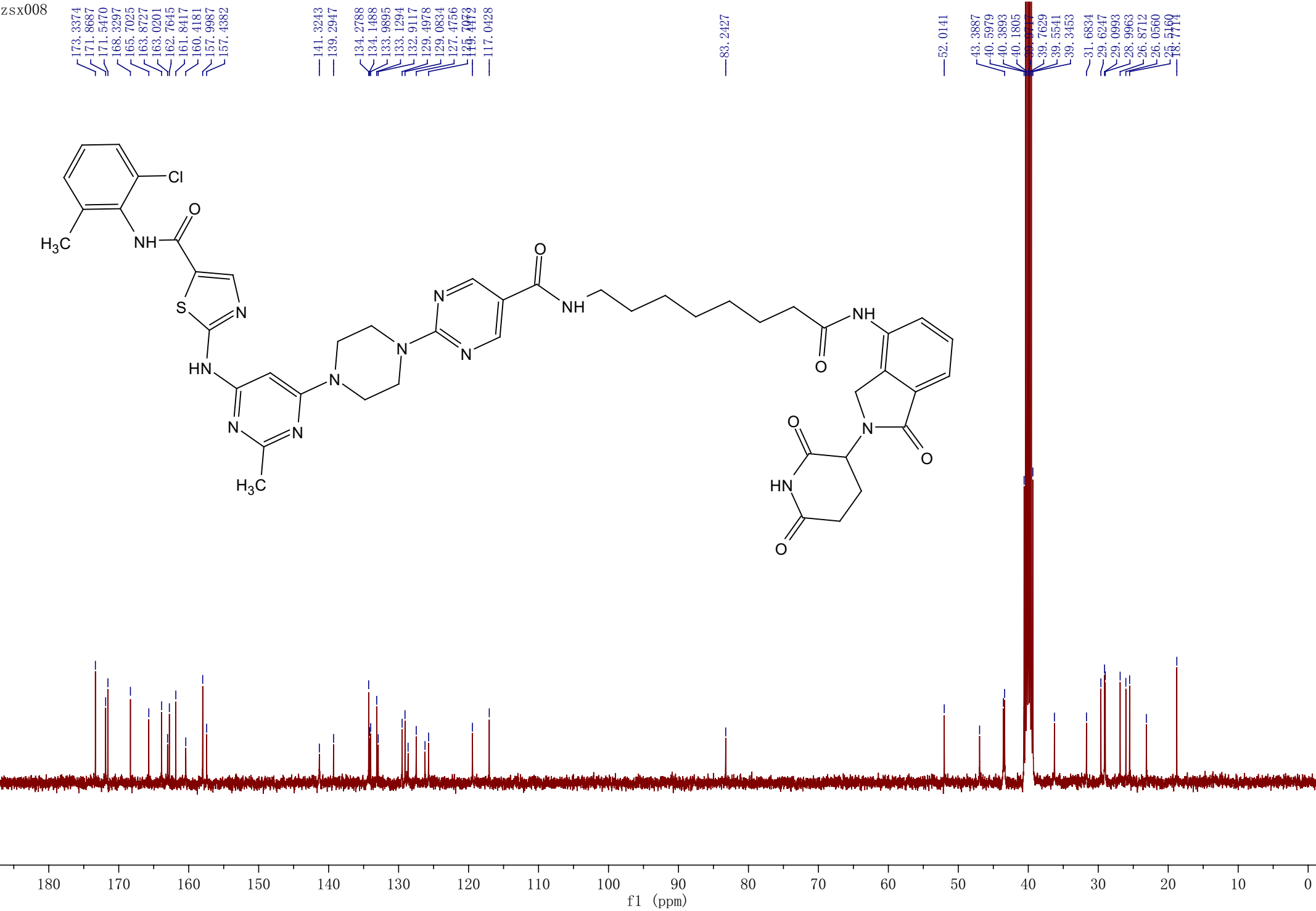

Supplement: Supplementary Materials — DMP6. 1H NMR (400 MHz, DMSO) δ 11.52 (s, 1H), 11.03 (s, 1H), 9.90 (s, 1H), 9.77 (s, 1H), 8.81 (s, 2H), 8.34 (t, J = 5.4 Hz, 1H), 8.24 (s, 1H), 7.82 (dd, J = 7.3, 1.4 Hz, 1H), 7.61-7.45 (m, 2H), 7.40 (dd, J = 7.3, 1.4 Hz, 1H), 7.32-7.13 (m, 2H), 6.12 (s, 1H), 5.16 (dd, J = 13.3, 5.1 Hz, 1H), 4.38 (q, J = 17.5 Hz, 2H), 3.93 (t, J = 5.3 Hz, 4H), 3.75-3.61 (m, 4H), 3.24 (dd, J = 12.6, 6.4 Hz, 2H), 2.99-2.83 (m, 1H), 2.68-2.56 (m, 1H), 2.45 (s, 3H), 2.40-2.30 (m, 3H), 2.25 (s, 3H), 2.03 (dd, J = 8.8, 3.7 Hz, 1H), 1.70 - 1.57 (m, 2H), 1.56-1.47 (m, 2H), 1.37-1.27 (m, 6H); 13C NMR (100 MHz, DMSO) δ 173.3, 171.9, 171.6, 168.3, 165.7, 163.9, 163.0, 162.7, 161.8, 160.4, 158.0, 157.4, 141.3, 139.3, 134.3, 134.2, 133.9, 133.1, 132.9, 129.5, 129.1, 128.6, 127.5, 126.2, 125.7, 119.5, 117.0, 83.2, 52.0, 46.9, 43.6, 43.3, 40.6, 40.3, 40.1, 36.2, 31.7, 29.6, 29.1, 29.0, 26.8, 26.1, 25.5, 23.1, 18.8. DMP7. 1H NMR (400 MHz, DMSO) δ 11.52 (s, 1H), 11.03 (s, 1H), 9.90 (s, 1H), 9.77 (s, 1H), 8.81 (s, 2H), 8.34 (t, J = 5.5 Hz, 1H), 8.24 (s, 1H), 7.82 (dd, J = 7.2, 1.6 Hz, 1H), 7.54-7.44 (m, 2H), 7.42-7.38 (m, 1H), 7.32-7.21 (m, 2H), 6.12 (s, 1H), 5.15 (dd, J = 13.3, 5.1 Hz, 1H), 4.37 (q, J = 17.5 Hz, 2H), 3.93 (d, J = 5.5 Hz, 4H), 3.72-3.63 (m, 4H), 3.25-3.16 (m, 2H), 3.00-2.84 (m, 1H), 2.65-2.57 (m, 1H), 2.44 (s, 3H), 2.40-2.32 (m, 3H), 2.25 (s, 3H), 2.09-1.98 (m, 1H), 1.64-1.56 (m, 2H), 1.53-1.46 (m, 2H), 1.29-1.21 (m, 18H); 13C NMR (100 MHz, DMSO) δ 173.3, 171.8, 171.5, 168.3, 165.6, 163.8, 163.0, 162.8, 161.8, 160.4, 158.0, 157.4, 141.3, 139.3, 134.3, 134.1, 134.0, 133.1, 129.3, 129.1, 127.5, 126.2, 125.7, 119.4, 117.0, 83.2, 52.0, 46.9, 43.5, 43.3, 36.3 31.6, 30.2, 29.6, 29.4, 29.2, 29.1, 26.9, 26.0, 25.6, 23.1, 22.6, 18.7, 14.4. DMP11.1H NMR (400 MHz, DMSO) δ 11.50 (s, 1H), 11.02 (s, 1H), 9.88 (s, 1H), 9.77 (s, 1H), 8.79 (s, 2H), 8.36 (t, J = 5.5 Hz, 1H), 8.24 (s, 1H), 7.81 (dd, J = 7.5, 1.2 Hz, 1H), 7.51 (dd, J = 7.5, 1.2 Hz, 1H), 7.49-7.44 (m, 1H), 7.39 (t, J = 7.4, 1.7 Hz, 1H), 7 [file 4056398.f1.zip › DMP-7-C NMR.pdf]

z.sx008

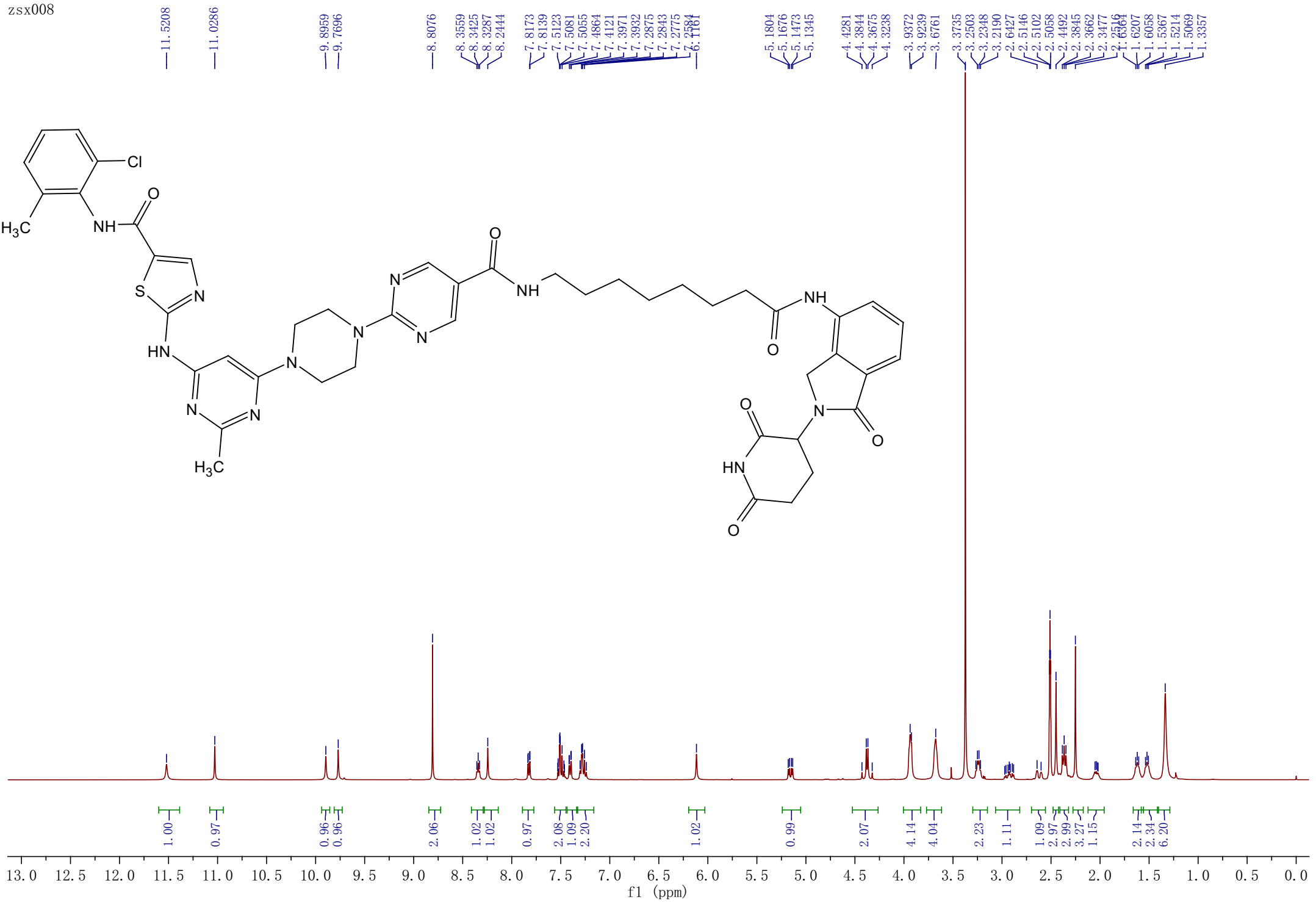

Supplement: Supplementary Materials — DMP6. 1H NMR (400 MHz, DMSO) δ 11.52 (s, 1H), 11.03 (s, 1H), 9.90 (s, 1H), 9.77 (s, 1H), 8.81 (s, 2H), 8.34 (t, J = 5.4 Hz, 1H), 8.24 (s, 1H), 7.82 (dd, J = 7.3, 1.4 Hz, 1H), 7.61-7.45 (m, 2H), 7.40 (dd, J = 7.3, 1.4 Hz, 1H), 7.32-7.13 (m, 2H), 6.12 (s, 1H), 5.16 (dd, J = 13.3, 5.1 Hz, 1H), 4.38 (q, J = 17.5 Hz, 2H), 3.93 (t, J = 5.3 Hz, 4H), 3.75-3.61 (m, 4H), 3.24 (dd, J = 12.6, 6.4 Hz, 2H), 2.99-2.83 (m, 1H), 2.68-2.56 (m, 1H), 2.45 (s, 3H), 2.40-2.30 (m, 3H), 2.25 (s, 3H), 2.03 (dd, J = 8.8, 3.7 Hz, 1H), 1.70 - 1.57 (m, 2H), 1.56-1.47 (m, 2H), 1.37-1.27 (m, 6H); 13C NMR (100 MHz, DMSO) δ 173.3, 171.9, 171.6, 168.3, 165.7, 163.9, 163.0, 162.7, 161.8, 160.4, 158.0, 157.4, 141.3, 139.3, 134.3, 134.2, 133.9, 133.1, 132.9, 129.5, 129.1, 128.6, 127.5, 126.2, 125.7, 119.5, 117.0, 83.2, 52.0, 46.9, 43.6, 43.3, 40.6, 40.3, 40.1, 36.2, 31.7, 29.6, 29.1, 29.0, 26.8, 26.1, 25.5, 23.1, 18.8. DMP7. 1H NMR (400 MHz, DMSO) δ 11.52 (s, 1H), 11.03 (s, 1H), 9.90 (s, 1H), 9.77 (s, 1H), 8.81 (s, 2H), 8.34 (t, J = 5.5 Hz, 1H), 8.24 (s, 1H), 7.82 (dd, J = 7.2, 1.6 Hz, 1H), 7.54-7.44 (m, 2H), 7.42-7.38 (m, 1H), 7.32-7.21 (m, 2H), 6.12 (s, 1H), 5.15 (dd, J = 13.3, 5.1 Hz, 1H), 4.37 (q, J = 17.5 Hz, 2H), 3.93 (d, J = 5.5 Hz, 4H), 3.72-3.63 (m, 4H), 3.25-3.16 (m, 2H), 3.00-2.84 (m, 1H), 2.65-2.57 (m, 1H), 2.44 (s, 3H), 2.40-2.32 (m, 3H), 2.25 (s, 3H), 2.09-1.98 (m, 1H), 1.64-1.56 (m, 2H), 1.53-1.46 (m, 2H), 1.29-1.21 (m, 18H); 13C NMR (100 MHz, DMSO) δ 173.3, 171.8, 171.5, 168.3, 165.6, 163.8, 163.0, 162.8, 161.8, 160.4, 158.0, 157.4, 141.3, 139.3, 134.3, 134.1, 134.0, 133.1, 129.3, 129.1, 127.5, 126.2, 125.7, 119.4, 117.0, 83.2, 52.0, 46.9, 43.5, 43.3, 36.3 31.6, 30.2, 29.6, 29.4, 29.2, 29.1, 26.9, 26.0, 25.6, 23.1, 22.6, 18.7, 14.4. DMP11.1H NMR (400 MHz, DMSO) δ 11.50 (s, 1H), 11.02 (s, 1H), 9.88 (s, 1H), 9.77 (s, 1H), 8.79 (s, 2H), 8.36 (t, J = 5.5 Hz, 1H), 8.24 (s, 1H), 7.81 (dd, J = 7.5, 1.2 Hz, 1H), 7.51 (dd, J = 7.5, 1.2 Hz, 1H), 7.49-7.44 (m, 1H), 7.39 (t, J = 7.4, 1.7 Hz, 1H), 7 [file 4056398.f1.zip › DMP-7-H NMR.pdf]
